# Alterations in gut and genital microbiota associated with gynecological diseases: a systematic review and meta-analysis

**DOI:** 10.1186/s12958-024-01184-z

**Published:** 2024-01-18

**Authors:** Ziwei Zhou, Yifei Feng, Lishan Xie, Song Ma, Zhaoxia Cai, Ying Ma

**Affiliations:** 1 Obstetrics and Gynecology Medical Center, Zhujiang Hospital, Southern Medical University, Guangzhou, China; 2 Guangzhou Liwan Maternal and Child Health Hospital, Guangzhou, China

**Keywords:** 16S rRNA sequencing, Diversity, Dysbiosis, Genital tract, Gut microbiome, Gynecological disease, Meta-analysis, Women’s health

## Abstract

**Background:**

Increasing number of studies have demonstrated certain patterns of microbial changes in gynecological diseases; however, the interaction between them remains unclear. To evaluate the consistency or specificity across multiple studies on different gynecological diseases and microbial alterations at different sites of the body (gut and genital tract), we conducted a systematic review and meta-analysis.

**Methods:**

We searched PubMed, Embase, Web of Science, and Cochrane Library up to December 5, 2022(PROSPERO: CRD42023400205). Eligible studies focused on gynecological diseases in adult women, applied next-generation sequencing on microbiome, and reported outcomes including alpha or beta diversity or relative abundance. The random-effects model on standardized mean difference (SMD) was conducted using the inverse-variance method for alpha diversity indices.

**Results:**

Of 3327 unique articles, 87 eligible studies were included. Significant decreases were found in gut microbiome of patients versus controls (observed species SMD=-0.35; 95%CI, -0.62 to -0.09; Shannon index SMD=-0.23; 95%CI, -0.40 to -0.06), whereas significant increases were observed in vaginal microbiome (Chao1 SMD = 1.15; 95%CI, 0.74 to 1.56; Shannon index SMD = 0.51; 95%CI, 0.16 to 0.86). Most studies of different diagnostic categories showed no significant differences in beta diversity. Disease specificity was observed, but almost all the changes were only replicated in three studies, except for the increased *Aerococcus* in bacterial vaginosis (BV). Patients with major gynecological diseases shared the enrichment of *Prevotella* and depletion of *Lactobacillus*, and an overlap in microbes was implied between BV, cervical intraepithelial neoplasia, and cervical cancer.

**Conclusions:**

These findings demonstrated an association between alterations in gut and genital microbiota and gynecological diseases. The most observed results were shared alterations across diseases rather than disease-specific alterations. Therefore, further investigation is required to identify specific biomarkers for diagnosis and treatment in the future.

**Supplementary Information:**

The online version contains supplementary material available at 10.1186/s12958-024-01184-z.

## Introduction

Gut microbiome, the “second genome” of human body, is the most abundant microbiome in the human body and most studied human microbiome that relates to obesity, inflammation, metabolism, cancer and so on [1–4]. Microbiota in the genital tract are mainly found in the lower genital tract (vagina, cervix) which has long been considered sterile [5]. Thanks to the rapid advancements of next-generation sequencing (NGS) technologies and bioinformatics, the understanding of microbiome colonized in various parts of human body is gradually improved, with more and more relative studies being carried out. Based on a recent study, the upper genital tract, including the uterus, fallopian tubes, and peritoneal fluid, harbors diverse communities of bacteria despite their low abundance [6]. However, very few studies could be retrieved due to the difficulty of invasive sampling of the upper reproductive tract.

Major gynecological diseases include bacterial vaginosis (BV), aerobic vaginitis (AE), vulvovaginal candidiasis (VVC), human papillomavirus infection (HPV), polycystic ovary syndrome (PCOS), endometriosis (EM), adenomyosis (AM), cervical intraepithelial neoplasia (CIN), cervical cancer (CCA), endometrial cancer (EC), uterine fibroids (UF) and ovarian cancer (OC) [7–10]. Systematic reviews in individual gynecological diseases have identified certain patterns of microbial changes. Women with EM had higher levels of *Proteobacteria, Enterobacteriaceae, Streptococcus* and *E. coli.* [11] More remarkably, patients with endometriosis have been found to have an increased *Firmicutes* to *Bacteroidetes* ratio, similar to irritable bowel syndrome [12]. Patients with PCOS had an increase in *Lactobacillus, Escherichia/Shigella* and *Bacteroides*, and a decrease in biodiversity in the gut microbiome [13]. *L. iners* was associated with higher HPV prevalence compared to *L. crispatus*, a protective factor in the progression from CIN to CCA [14, 15]. Another study demonstrated the protective effect of each *Lactobacillus* species against vaginal dysbiosis, as well as a strong probiotic multi-microbial consortium by *L. iners*, *L. jensenii*, *L. gasseri*, and *L. acidophilus* against AV and BV [16]. However, the specificity and reproducibility of the microbial changes among different diseases require further exploration because of the inconsistent results from the individual studies. Consequently, it is crucial to characterize the microbial diversity and composition across a broader range of gynecological diseases.

Our aim was to discover the bidirectional interaction between gynecological diseases and microbial alterations at different sites of the body (gut and genital tract) and to evaluate the consistency or specificity across multiple studies focused on gynecological diseases in adult women, which in turn may provide potential biomarkers. Therefore, in the present, we conducted a systematic review and meta-analysis of studies that characterized the composition of the microbiota between women with and without gynecological diseases. In addition to the studies on the intestinal tract, those on the genital tract were also included.

## Methods

The protocol of study was preregistered with PROSPERO (CRD42023400205). We also followed the Preferred Reporting Items for Systematic Reviews and Meta-analyses (PRISMA) reporting guideline [17].

### Search strategy and information sources

The search was conducted using PubMed, Embase, Web of Science, and Cochrane Library and was last updated on December 5, 2022. The search strings have been presented in Appendix S1. Included studies were limited to those including human studies and were published in English since 2005. The reference lists of relevant reviews were also manually examined to identify additional eligible studies missed by the initial search.

### Eligibility criteria and study selection

Studies were eligible if they [1] focused on major gynecological diseases in adult women as previous described in introduction, [2] applied next-generation sequencing on the microbiome from the intestinal tract, genital tract, peritoneal fluid, or biopsy sample, and [3] reported outcomes including alpha or beta diversity or relative abundance. Interventional studies and studies without control groups were excluded. Two reviewers independently screened titles and abstracts. A full-text assessment was then performed by them. Discrepancies were resolved by discussion with a third author or among all reviewers.

### Data extraction and assessment of risk of bias

A pilot-tested form was used to extract data from eligible studies. For each study, the following information was extracted: first author’s name, year of publication, country, number of patients and controls, age and Body Mass Index (BMI) of participants, sampling type, sequencing platform, and database. As for primary outcomes, we extracted the alpha and beta diversity of microbial communities, as well as the relative abundance of taxonomic findings at the phylum, family, and genus levels.

The quality of included studies was examined independently by two reviewers using the Newcastle–Ottawa Scale (NOS) for case–control studies [18]. The NOS is based on three dimensions: selection, comparability, and outcome. A total NOS score of ≤ 5 was considered low-quality.

### Data synthesis

For studies that did not provide original data but box plots of alpha diversity indices, we used WebPlotDigitizer version 4.6 to extract the data from the figures according to previously published methods [19]. The sample mean and standard deviation were estimated using a web-based tool (https://www.math.hkbu.edu.hk/~tongt/papers/median2mean.html) with the sample size, median, range, and interquartile range [20]. If the data were significantly skewed, an alternative validated procedure was followed [21]. In consideration of the high likelihood of between-study differences, the random-effects model on standardized mean difference (SMD) was conducted using the inverse-variance method and were visualized by means of forest plots. Tests for heterogeneity were reported using the I^2^ statistic, which was categorized as low (25-50%), moderate (51-75%), or high (> 75%). Publication bias was examined with funnel plots. Preplanned subgroup analyses were performed by specific type of gynecological disease. In addition, further subgroup analyses were performed by country and body weight of participants in studies on PCOS, as there are sufficient studies for such a classification. All statistical analyses were conducted in Review Manager version 5.4 for Windows (Cochrane Collaboration).

## Results

### Study selection

**Fig. 1:**
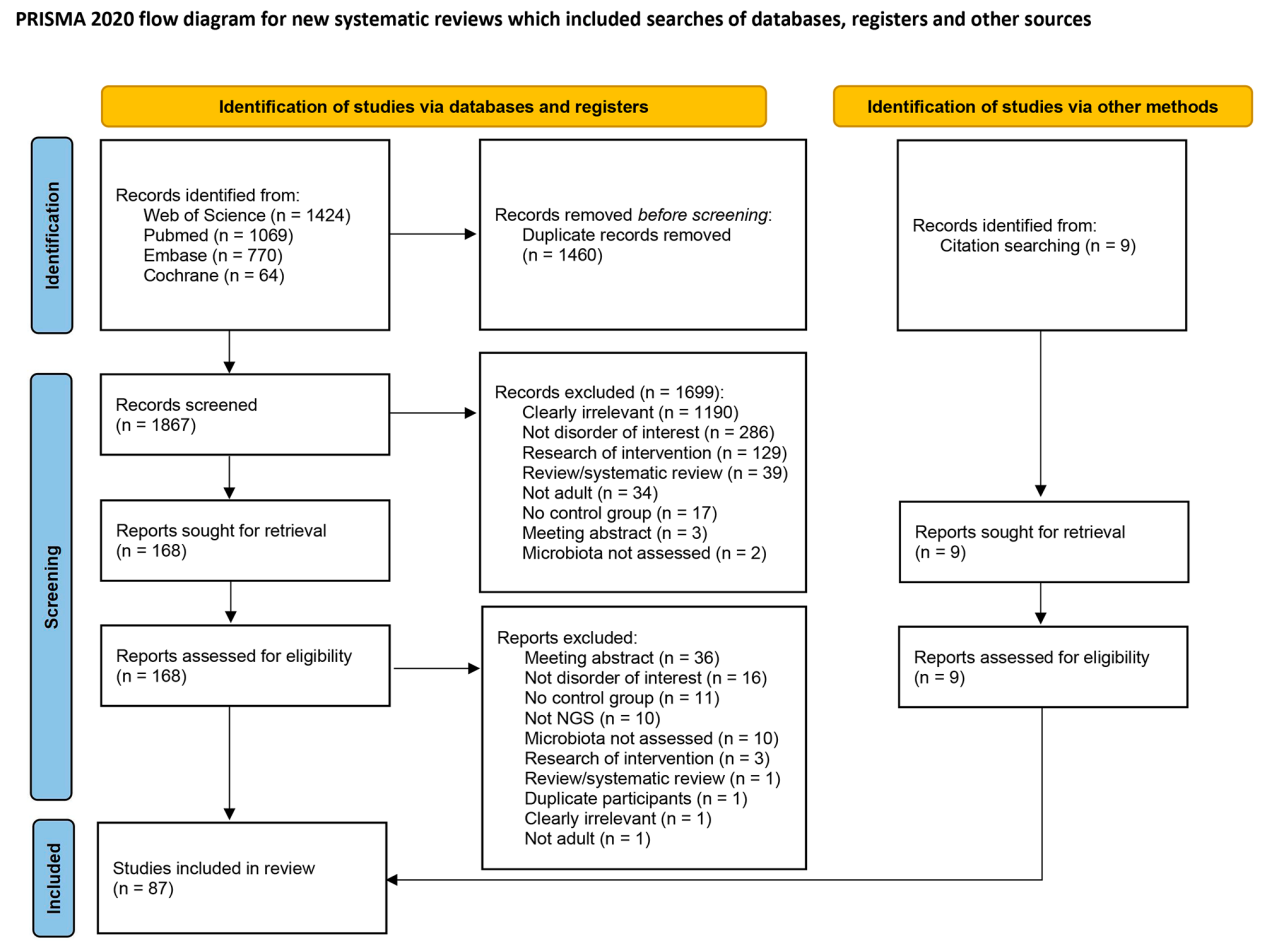
PRISMA flowchart showing the study selection process

A total of 3327 articles were identified through the preliminary search. After removing duplicates, 1876 articles were screened based on the titles and abstracts. A total of 177 full-text articles were retrieved for a detailed eligibility evaluation, 90 of which were excluded. As a result, 87 studies across nine diseases were included in our meta-analysis (Fig. 1). The number of studies included for each disease was 13 for BV, 13 for EM, one for AM, 26 for PCOS, 10 for CIN, 14 for CCA, four for OC, five for EC, and one for UF.

### Study characteristics and methodologies

Table S1 describes the characteristics of the included studies. In total, 7726 participants were included, and the sample sizes of the included studies ranged from 13 to 439. Most studies (n = 58) were conducted in East Asia (45 in China), 12 in Europe, 14 in North and South America, one in Africa, and two in multiple countries.

The methodology of composition analysis and sequencing details were various, with 16 S rRNA sequencing being most common (Table S2). We also noted heterogeneity regarding the amplified region of the 16 S rDNA, with the most widely used being V3-V4 (n = 37 studies), and V4 (n = 20). Most of these sequencing analyses were conducted on the Illumina MiSeq, Illumina HiSeq, Ion PGM, and 454 GS platforms. The remaining three studies used shotgun metagenomics and created metagenome libraries. The most widely used databases were the Greengenes database and Ribosomal Database Project (RDP) Classifier. Besides, most studies (76/87) were matched by age, some of which also carried out subgroup analyses stratified by BMI, stage of disease, HPV status, smoking, or other disease-related factors.

### Risk of bias of included studies

As shown in Table S3, all of the included studies were assessed using the Newcastle-Ottawa Scale (NOS) and received a moderate score ranging from 6 to 8 (mean = 7.10). None of the included studies scored in the non-response rate category, which is not applicable to these study methodologies. No study was excluded because of the risk of bias (Table S3).

### Alpha diversity

79 of the 87 studies assessed the alpha diversity of the microbial communities, among which 64 provided statistical charts or accurate data between the patients and controls. Within each sample type, indices with sufficient studies (n > 5) were included in the meta-analysis. The most widely used indices were the Shannon index, Chao1, observed species, and Simpson index. Visual inspection revealed no evidence of publication bias in any of the funnel plots (Figure S1).

**Fig. 2:**
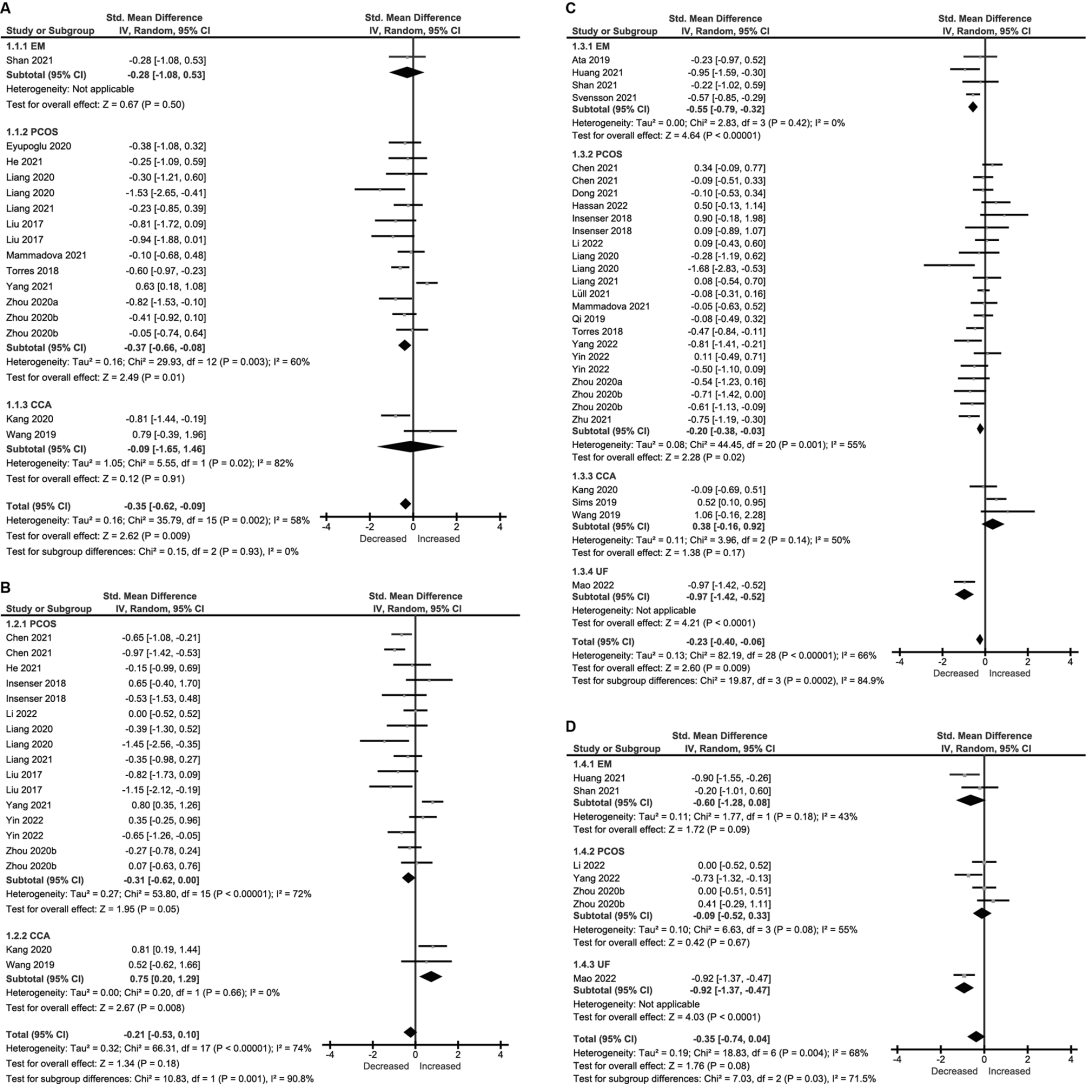
Forest Plots of Alpha Diversity in the Gut Microbiota of Patients with Gynecological Diseases Compared with Healthy Controls. (A) Observed species. (B) Chao1. (C) Shannon index. (D)Simpson index

Regarding the richness of the gut microbiome, 13 studies reported observed species in patients (n = 366) and controls (n = 283) [22–34]. A significant decrease in patients (standardized mean difference [SMD]=-0.35, [95%CI: -0.62, -0.09], I^2^ = 58%) was observed when pooling the data during the meta-analysis (Fig. 2A). Within individual diagnoses, there was a significant decrease in PCOS. Twelve studies reported Chao1 in patients (n = 413) and controls (n = 331), with no statistically significant change in the pooled estimate (SMD=-0.21, [95%CI: -0.53, 0.10], I^2^ = 74%) (Fig. 2B) [24–27, 30, 32–38].

Regarding the diversity of the gut microbiome, 24 studies reported the Shannon index in patients (n = 929) and controls (n = 1042) [22, 25, 26, 28, 29, 31–49]. There was a significant decrease in patients (SMD=-0.23, [95%CI: -0.40, -0.06], I^2^ = 66%) (Fig. 2C). Considering individual diagnoses, there was a significant decrease only in EM and PCOS. Six studies reported Simpson in patients (n = 156) and controls (n = 118), and no significant difference between groups was observed (SMD=-0.24, [95%CI: -0.62, 0.15], I^2^ = 57%) (Fig. 2D) [22, 32, 37, 40, 46, 49].

To understand inter-study heterogeneity, we further performed a subgroup analysis according to the BMI and country of participants for sufficient studies in PCOS (Table S4). It should be noted that BMI may have an association with findings. Decreases in alpha diversity indices were consistently seen in obese PCOS patients and heterogeneity between studies was substantially reduced to 0%. We noticed that the majority of studies were conducted in China, suggesting whether the findings were influenced by dietary structures between different countries. All three indices showed different outcomes in China compared with others, with moderate heterogeneity still observed.

**Fig. 3:**
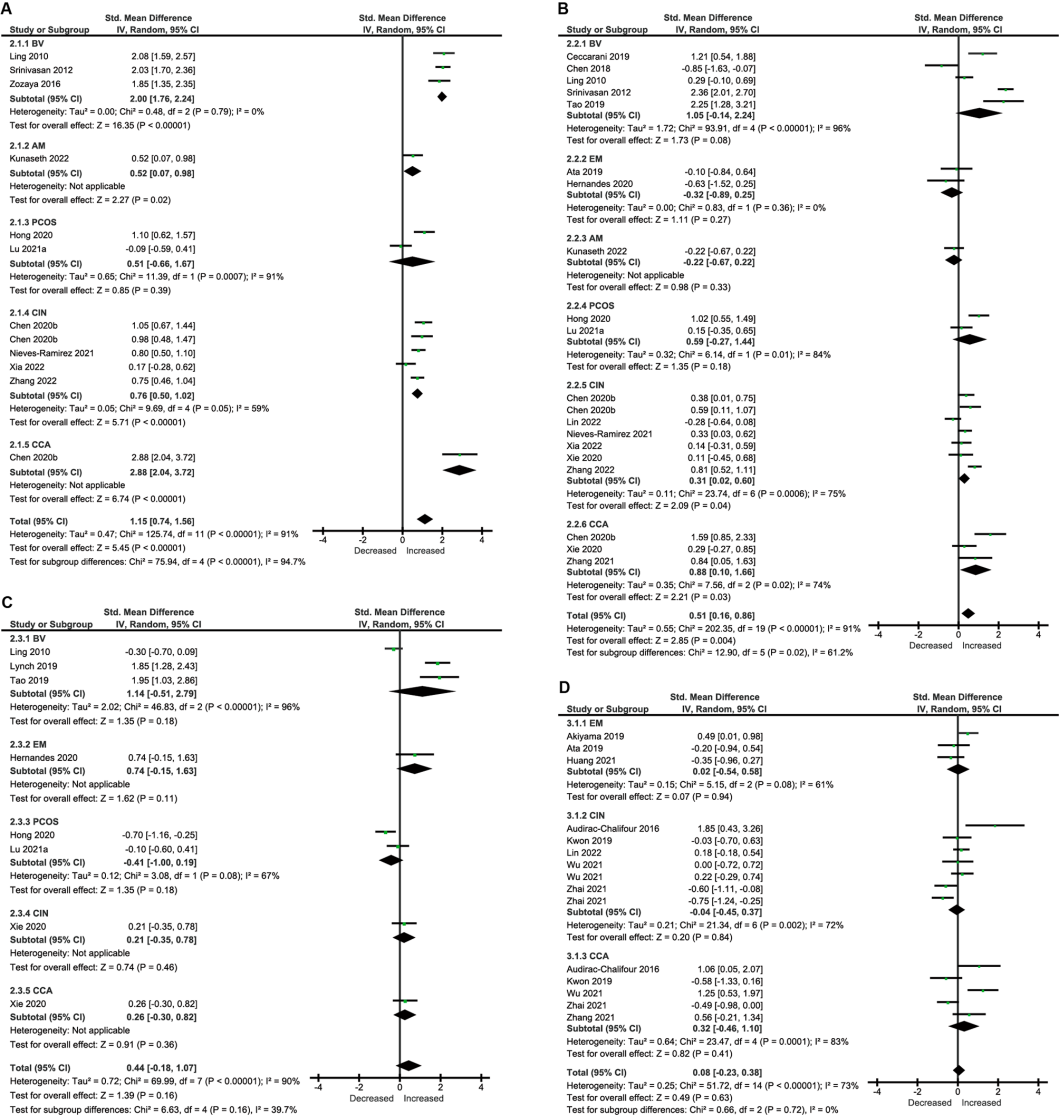
Forest Plots of Alpha Diversity in the Genital Microbiota of Patients with Gynecological Diseases Compared with Healthy Controls. (A to C) Alpha Diversity in the vaginal microbiota (A) Chao1. (B) Shannon index. (C) Simpson index. (D) Shannon index in the cervical microbiota

With regard to vaginal microbiome composition, 10 studies provided data on Chao1 in patients (n = 656) versus controls (n = 736), and there was a significant increase in patients (SMD = 1.15, [95%CI: 0.74, 1.56], I^2^ = 91%) (Fig. 3A) [50–59]. Data on Shannon index were reported by 17 studies in patients (n = 771) versus controls (n = 925) [39, 50, 51, 53–66]. The pooled estimate demonstrated a significant increase in patients (SMD = 0.51, [95%CI: 0.16, 0.86], I^2^ = 91%) (Fig. 3B). Seven studies provided data on Simpson in patients (n = 231) vs. controls (n = 233), and the difference between them was not significant (SMD = 0.44, [95%CI: -0.18, 1.07], I^2^ = 90%) (Fig. 3C) [50, 54, 55, 62, 63, 65, 67]. Within diagnostic categories, those increases were mainly seen in CIN and CCA with moderate to high heterogeneity.

As for cervical microbiota, nine studies provided data on the Shannon index in patients (n = 81) versus controls (n = 105) [39, 40, 64, 66, 68–72]. The pooled data showed no significant differences (SMD = 0.08, [95%CI: -0.23, 0.38], I^2^ = 66%) (Fig. 3D). Only four studies provided data on Simpson, which were not included in the meta-analysis [40, 70–72].

### Beta diversity

Beta diversity compares the differences in microbial community composition between samples from different body sites in patients and controls. 68 of the 87 studies conducted beta diversity using variable measures (Table S5). Regarding fecal microbiota, 30 studies assessed the beta diversity between patients and controls. Among them, 3 of 5 studies in EM, 4 of 21 in PCOS, and 1 of 3 in cervical cancer reported significant differences. In the vaginal microbiota, 4 of 5 studies in BV showed significant differences between groups, and one study showed consistently significant difference of beta diversity between groups with HPV infection, cervical dysplasia, and cervical cancer. In the cervical microbiota, consistent no significant differences were reported by all three studies in EM and one in PCOS. Because of the different grades of lesions in patients with CIN and cervical cancer, the beta diversity of the different studies could not be combined for analysis. All of the two studies in ovarian tissue and more than half of the studies of peritoneal fluid reported significant differences, while most of the studies of endometrial tissue showed no significant difference.

### Differentially abundant microbial taxa

**Fig. 4:**
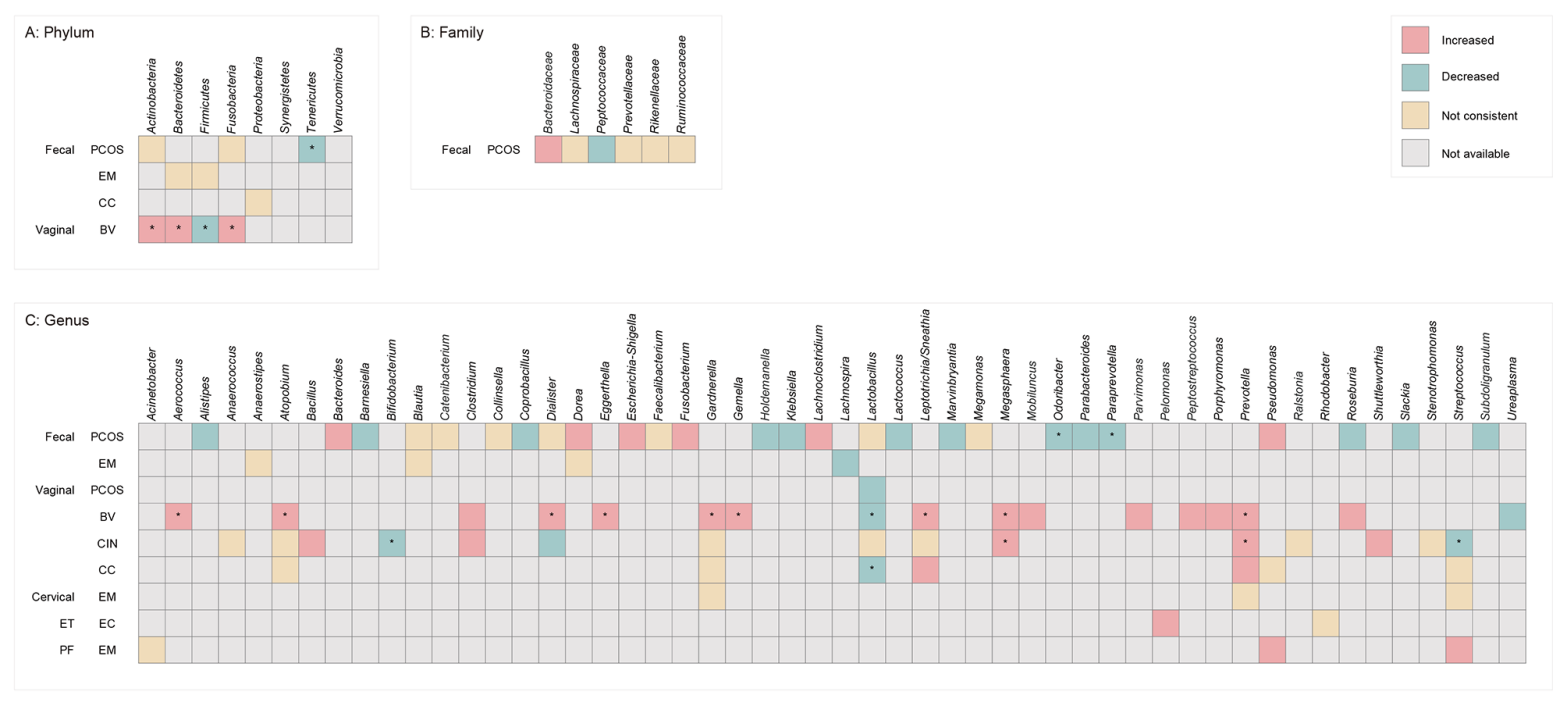
Summary of Changes in Relative Abundance of Microbial Taxa (Phylum, Family, Genus) by at Least 2 Studies from a Diagnostic Category. ET, endometrial tissue; PF, peritoneal fluid; * consistent in 3 or more studies

We summarized the representative taxa at three levels (phylum, family, and genus). Seventy of the 87 studies assessed the relative abundance of microbial taxa between patients and control groups. Overall, 22 phyla, 50 families, and 154 genera were identified from six different sample types, including stool, vaginal swab, cervical mucus, endometrial tissue (ET), ovarian tissue (OT), and peritoneal fluid (PF). To summarize within- and between- disease comparisons for the microbial taxa, in studies reporting the same microbes, findings with less than 60% agreement were categorized as “not consistent” (Fig. 4).

#### Disease specificity

If a taxon was altered in the same direction in only a single disease and was consistent in more than two studies, it was considered a candidate for disease specificity. Thus, our findings indicate some disease-specific alterations, such as the depletion of *Odoribacter* and *Paraprevotella* in PCOS. We also observed a depletion of *Bifidobacterium* in CIN. There was also evidence of the enrichment of *Aerococcus*, *Eggerthella* and *Gemella* in BV. However, there was limited evidence of these disease specificities because the majority of the consistent within-disease alterations were reproduced in only three studies.

#### Shared alteration across diseases

Our study indicates a consistent alteration across diseases for certain microbes. The most consistent changes were the enrichment of *Prevotella* (PCOS, BV, and CC) and the depletion of *Lactobacillus* in three diseases (PCOS, BV, and CC). The *Clostridium* and *Megasphaera* genera were enriched in both BV and CIN, whereas *Sneathia* was enriched in both BV and CC. Therefore, an overlap between BV, CIN, and CC was implied in the vaginal microbiota.

## Discussion

This meta-analysis assessed microbiota alterations with the aim of evaluating consistency or specificity across a spectrum of gynecological diseases. We summarized the alpha diversity (within sample), beta diversity (between samples), and differentially abundant microbial taxa at the phylum, family, and genus levels. The main findings were as follows: [1] a small effect size decrease in observed species and Shannon index of gut microbiota; a moderate to large effect size increase in Chao1 and Shannon index of vaginal microbiota; [2] significant differences of β diversity were reported mostly in EM and BV, whereas no significant differences were frequently reported; [3] disease specificity was observed in three diseases, but almost all the changes were only replicated in three studies, except for *Aerococcus* in BV; [4] patients with major gynecological diseases shared the general changes of the enrichment of *Prevotella* and the depletion of *Lactobacillus* in vaginal samples; an overlap was implied between BV, CIN, and CC.

In terms of alpha diversity, our meta-analysis showed that both richness and diversity of the gut microbiota in patients with gynecological diseases showed a statistically significant decrease. In terms of individual diagnosis, the Shannon index of gut microbiota was significantly decreased in EM patients with no heterogeneity between studies. In previous animal experiments, EM mice showed a significant decrease in Shannon index and metabolites like alpha-linolenic acid, which can increase the expression of the ZO-2 protein in the intestinal wall, reduce the content of LPS and the aggregation of macrophages in the peritoneum, as well as decreased the oxidative stress and inflammation [73–75]. However, Yuan et al. [76] reported no significant difference in Shannon index. The pooled estimate of our meta-analysis favored a reduction in the diversity of the intestinal flora of the EM patients, in accordance to the hypothesis that diverse communities may increase the stability and productivity of an ecosystem. Moreover, antibiotic-induced microbiota-depleted mice has shown a reduction of endometriotic lesion, possibly by fecal metabolite like quinic acid and modulation of immune cell populations like macrophages in peritoneum [77].

On the other hand, the observed species and Shannon index in PCOS patients were significantly decreased. The subgroup analysis on their BMI demonstrated that observed species, Chao1, and Shannon index were significantly decreased in obese PCOS patients with 0-13% heterogeneity, but no significant differences were shown of Chao1 and Shannon in non-obese patients. Obesity is one of the representative features of PCOS, which was associated with lower alpha diversity of the gut microbiome in previous meta-analyses [78–80]. As we suspected, metabolism and obesity have specific interactions with the gut microbiome of PCOS, but the mechanism remains to be further demonstrated. Generally, gut microbiome may contributed to PCOS by affecting energy absorption, short chain fatty acids, lipopolysaccharide, choline and bile acid, intestinal permeability and the brain-gut axis [81]. Meanwhile, in the reproductive endocrine system, the gut microbiome has a complex interaction with insulin, estrogen, and testosterone. The intestinal flora promotes the glucose metabolism disorder of PCOS possibly through the FXR signaling pathway, while the removement of it decreases serum testosterone levels, ameliorated insulin resistance and increased relative FXR mRNA levels [30]. Microbiome is also affected by genetic, nutritional, and environmental factors, resulting in a high heterogeneity among individuals [9]. Through meta-analysis and subgroup analysis, we tried to minimize the effect of heterogeneity among studies and obtained more trustworthy pooled results.

Vaginal microbiota dysbiosis, characterized by a progressive replacement of certain *Lactobacillus* species by pathogenic microorganisms who can develop biofilms, plays an important role in gynecological diseases [82, 83]. Analysis showed that Chao1 and Shannon indices were significantly increased in patients, mainly in CIN and CCA, which proved that vaginal dysbiosis was associated with an increased risk of persistent HPV infection and cervical dysplasia, as previous studies carried out [14, 15, 84, 85]. Meanwhile, no significant differences were observed in cervical microbiota.

In our meta-analysis, we found that studies related to upper genital tract remain very few. A recent study revealed that intracellular microbiota in breast tumor tissue regulated the host-cell viability and metastatic colonization through reorganizing actin cytoskeleton and enhancing resistance to fluid shear stress [86]. Another study has demonstrated the microbial continuum in the female reproductive tract, with different microbial communities distributed from lower to upper genital tract. ET and PF samples contains lower bacterial biomass but higher diversity than vaginal sites, which is similar to tumor tissue [6]. These findings give us a hint that future studies can investigate more about the microbiota in tissues and its mechanism to uterine-related diseases such as EM, EC, and OC.

As for beta diversity, most studies of different diagnostic categories and sites demonstrated no significant differences between patients and controls, or conflicting results between studies. No consistent changes were observed, suggesting that the measurement of beta diversity may not be suitable as a diagnostic biomarker.

In general, disease specificity was observed in PCOS, BV, and CIN, but these alterations were weakly reproduced because most of the consistent within-disease changes were replicated in only three studies. It is worth noting that the *Aerococcus* genus was enriched in four of seven studies on BV (Figure S2D), which can also cause urinary tract infections and infective endocarditis [87, 88]. Furthermore, the *Odoribacter* genus was decreased in PCOS, which is a promising probiotic for its ability to improve glucose tolerance and inflammation related to obesity [89]. Researchers have found that the trypsin-degrading property is conserved in all *Paraprevotella* strains, which were also decreased in PCOS [90]. In addition, the *Streptococcus* genus, a pro-inflammatory microbe, was increased in the fecal and peritoneal fluid of EM patients, which is consistent with a previous meta-analysis reporting its enrichment in cervical mucus and endometrial tissue [11]. This indicated the migration of microbes along the genital tract, thus contributing to the imbalance of bacteria in the peritoneal fluid.

Our findings indicate shared alterations of vaginal microbial changes between BV, CIN, and CC, as mentioned before. Most consistently in vaginal microbiota, the *Clostridium*, *Sneathia*, *Megasphaera*, and *Prevotella* genera were enriched, while the *Lactobacillus* genus was depleted. A study on *Prevotella* reported its relation with the production of sialidase, an enzyme that enhances the ability of microorganisms to invade and destroy tissue [91]. Candidate gene analysis also showed associations between genetic variants of interleukin-5 and the abundance of *Prevotella* spp [92]. Recently, another study suggested that *Prevotella* was significantly correlated with the expression levels of NF-κB and C-myc in cervical cells, influencing the occurrence of HPV infection and cervical lesions [93].

### Strengths and limitations

To the best of our knowledge, this is the first meta-analysis that integrates microbiota alterations across the wide range of gynecological diseases. However, the limitations of this study must be mentioned. First, the modest sample sizes of some included studies and the small number of studies on the upper genital tract may have undermined the reliability of the related results, requiring further validation with larger sample sizes. Second, inter-study heterogeneity was observed, especially in vaginal samples, which makes the findings difficult to replicate and the quality of the evidence reported low. Additional subgroup analyses are required to explore the sources of heterogeneity. Third, because of the exclusion of studies with interventions, we were unable to explore the confounding effects of antibiotics or other treatments, since we know antimicrobial resistance has been more frequent especially in BV [94]. Finally, our study focused on microbial composition rather than function. The role of gut and genital microbiota in gynecological diseases should be further elucidated by functional analysis.

## Conclusion

Our study demonstrated that alterations in gut and genital microbiota are associated with major gynecological diseases. The overall results showed a decrease of diversity in gut microbiota and an increase in genital tract. The most observed results were shared alterations across diseases rather than disease-specific alterations. Therefore, further investigation is required to identify specific biomarkers that may serve as promising diagnostics in the future and to explore the mechanisms and pathways between gynecological diseases and microbial alterations.

### Electronic supplementary material

Below is the link to the electronic supplementary material.

Supplementary Material 1

## Data Availability

The authors confirm that the data are available within the article, supplementary materials, and referenced literature, from which the data were extracted.
