# Supplementary material for: Alterations in gut and genital microbiota associated with gynecological diseases: a systematic review and meta-analysis
Source: Reprod Biol Endocrinol. 2024 Jan 18;22:13. doi: 10.1186/s12958-024-01184-z (PMC10795389; doi:10.1186/s12958-024-01184-z)
Supplement: Supplementary file 1 — Supplementary Material 1 [file 12958_2024_1184_MOESM1_ESM.docx]

Contents

- Appendix S1. Electronic search strategy in Cochrane Library databases, PubMed, Web of Science and Embase
- Table S1. Detailed characteristics of the included studies
- Table S2. Methodology of composition analysis and sequencing details
- Table S3. Quality Assessment of the Included Studies in Meta-analysis Using the Newcastle-Ottawa Scale
- Table S4. Subgroup analyses of studies with PCOS patients on different regions and weight (Fecal samples)
- Table S5. Methodology and findings of the included studies assessing beta diversity
- Figure S1. Funnel plots assessing publication bias in the meta-analyses of alpha diversity
- Figure S2. Figures for study-level findings of relative abundance of gut and genital microbes

Appendix S1. Electronic search strategy in Cochrane Library databases, PubMed, Web of Science and Embase

**1.1 Cochrane Library**

| ID | Search | Hits |
| --- | --- | --- |
| #1 | Gynaecology:tp (Word variations have been searched) | 587 |
| #2 | (microbiome) OR (microbiota) OR (ecosystem) OR (bacteria) OR (flora) OR (microflora) OR (dysbiosis) | 26031 |
| #3 | (gut) OR (gastrointestinal) OR (intestinal) OR (feacal) OR (fecal) OR (stool) OR (feces) | 106198 |
| #4 | (genital tract) OR (vaginal) OR (cervix mucus) OR (cervical mucus) OR (cervical swab) | 23218 |
| #5 | (ovarian tissue) OR (ovarian cancer tissue) OR (ovarian surface tissue) OR (ovarian biopsy) OR (ovarian sample) | 2986 |
| #6 | (endometrial tissue) OR (endometrial biopsy) OR (endometrial sample) OR (endometrium tissue) OR (endometrium biopsy) OR (endometrium sample) | 2584 |
| #7 | (Peritoneal Fluid) OR (Ascitic Fluid) OR (peritoneal effusion) | 1358 |
| #8 | #1 AND #2 AND #3 in Cochrane Reviews | 19 |
| #9 | #1 AND #2 AND #4 in Cochrane Reviews | 26 |
| #10 | #1 AND #2 AND #5 in Cochrane Reviews | 9 |
| #11 | #1 AND #2 AND #6 in Cochrane Reviews | 8 |
| #12 | #1 AND #2 AND #7 in Cochrane Reviews | 2 |

**1.2 PubMed**

| ID | Search | Hits |
| --- | --- | --- |
| #1 | ((gut) OR (gastrointestinal) OR (intestinal) OR (feacal) OR (fecal) OR (stool) OR (feces)) |  |
| #2 | ((genital tract) OR (vaginal swab) OR (vaginal) OR (cervix mucus) OR (cervical mucus) OR (cervical swab)) |  |
| #3 | ((endometrial tissue) OR (endometrial biopsy) OR (endometrial sample) OR (endometrium tissue) OR (endometrium biopsy) OR (endometrium sample)) |  |
| #4 | ((ovarian) OR (ovary)) AND ((tissue) OR (biopsy) OR (sample)) |  |
| #5 | ((Peritoneal Fluid) OR (Ascitic Fluid) OR (peritoneal effusion)) |  |
| #6 | ((microbiome) OR (microbiota) OR (ecosystem) OR (bacteria) OR (flora) OR (microflora) OR (dysbiosis)) |  |
| #7 | ((16S) OR (Sequencing) OR (Shotgun) OR (Metagenome) OR (NGS) OR (illumina) OR (pyrosequencing)) |  |
| #8 | ((Endometriosis) OR (Endometrioma) OR (Endometrioses)) |  |
| #9 | ((Leiomyoma) OR (Fibromyoma) OR (Fibroid Uterus) OR (Uterine Fibroma) OR (Uterine Fibroid) OR (Adenomyosis) OR (Adenomyoses) OR (Endometritis) OR (Endomyometritis)) |  |
| #10 | ((Polycystic Ovary Syndrome) OR (Polycystic Ovarian Syndrome) OR (Stein Leventhal Syndrome)) |  |
| #11 | ((Bacterial Vaginosis) OR (Bacterial Vaginitides) OR (Bacterial Vaginoses) OR (Bacterial Vaginitis)) |  |
| #12 | ((Cervical Intraepithelial Neoplasia) OR (Cervical Intraepithelial Neoplasm) OR (Squamous Intraepithelial Lesion) OR (Cervical Cancer) OR (Cervix Cancer)) |  |
| #13 | ((Endometrial Cancer) OR (Endometrial Carcinoma) OR (Endometrium Carcinoma) OR (Endometrium Cancer)) |  |
| #14 | ((ovarian cancer) OR (ovary cancer)) |  |
| #15 | ((humans[Filter]) AND (2005:2022/12/5[pdat]) AND (english[Filter])) |  |
| #16 | #1 AND #6 AND #7 AND #8 AND #15 | 13 |
| #17 | #1 AND #6 AND #7 AND #9 AND #15 | 3 |
| #18 | #1 AND #6 AND #7 AND #10 AND #15 | 38 |
| #19 | #1 AND #6 AND #7 AND #11 AND #15 | 33 |
| #20 | #1 AND #6 AND #7 AND #12 AND #15 | 45 |
| #21 | #1 AND #6 AND #7 AND (#13 OR #14) AND #15 | 34 |
| #22 | #2 AND #6 AND #7 AND #8 AND #15 | 11 |
| #23 | #2 AND #6 AND #7 AND #9 AND #15 | 16 |
| #24 | #2 AND #6 AND #7 AND #10 AND #15 | 6 |
| #25 | #2 AND #6 AND #7 AND #11 AND #15 | 343 |
| #26 | #2 AND #6 AND #7 AND #12 AND #15 | 292 |
| #27 | #2 AND #6 AND #7 AND (#13 OR #14) AND #15 | 20 |
| #28 | #3 AND #6 AND #7 AND #13 AND #15 | 57 |
| #29 | #4 AND #6 AND #7 AND #14 AND #15 | 149 |
| #30 | #5 AND #6 AND #7 AND (#8 OR #12 OR #13 OR #14) AND #15 | 9 |

**1.3 Web of Science**

| ID | Search | Hits |
| --- | --- | --- |
| #1 | ((TS=(gut)) OR (TS=(gastrointestinal)) OR (TS=(intestinal)) OR (TS=(feacal)) OR (TS=(fecal)) OR (TS=(stool)) OR (TS=(feces))) |  |
| #2 | ((TS=(genital tract)) OR (TS=(vaginal swab)) OR (TS=(vaginal)) OR (TS=(cervix mucus)) OR (TS=(cervical mucus)) OR (TS=(cervical swab))) |  |
| #3 | ((TS=(Peritoneal Fluid)) OR (TS=(Ascitic Fluid)) OR (TS=(peritoneal effusion))) |  |
| #4 | ((TS=(endometrial tissue)) OR (TS=(endometrial biopsy)) OR (TS=(endometrial sample)) OR (TS=(endometrium tissue)) OR (TS=(endometrium biopsy)) OR (TS=(endometrium sample))) |  |
| #5 | ((TS=(ovarian tissue)) OR (TS=(ovarian cancer tissue)) OR (TS=(ovarian surface tissue)) OR (TS=(ovarian biopsy)) OR (TS=(ovarian sample))) |  |
| #6 | ((TS=(microbiome)) OR (TS=(microbiota)) OR (TS=(ecosystem)) OR (TS=(bacteria)) OR (TS=(flora)) OR (TS=(microflora)) OR (TS=(dysbiosis))) |  |
| #7 | ((TS=(16s)) OR (TS=(sequencing)) OR (TS=(shotgun)) OR (TS=(metagenome)) OR (TS=(NGS)) OR (TS=(illumina)) OR (TS=(pyrosequencing))) |  |
| #8 | ((TS=(endometrios*s)) OR TS=(endometrioma*)) |  |
| #9 | ((TS=(Polycystic Ovary Syndrome)) OR (TS=(Polycystic Ovarian Syndrome)) OR (TS=(Stein Leventhal Syndrome)) OR (TS=(Sclerocystic Ovarian Degeneration)) OR (TS=(Sclerocystic Ovary Syndrome))) |  |
| #10 | ((TS=(Leiomyoma*)) OR (TS=(Fibroid Tumor*)) OR (TS=(Fibromyoma*)) OR (TS=(Fibroid Uterus)) OR (TS=(Uterine Fibroid*)) OR (TS=(Uterine Fibroma*)) OR (TS=(Adenomyos*s)) OR (TS=(Endometritis)) OR (TS=(Endomyometritis))) |  |
| #11 | (((TS=(Bacterial Vaginitides)) OR TS=(Bacterial Vaginos*s)) OR TS=(Bacterial Vaginitis)) |  |
| #12 | ((TS=(Cervical Intraepithelial Neoplasia)) OR TS=(Cervical Intraepithelial Neoplasm*) OR (TS=(Uterine Cervical Neoplasm*)) OR (TS=(Cervical Neoplasm*)) OR (TS=(Cervix Neoplasm*)) OR (TS=(Cancer of the Uterine Cervix)) OR (TS=(Cancer of the Cervix)) OR (TS=(Cervical Cancer)) OR (TS=(Uterine Cervical Cancer*)) OR (TS=(Cancer of Cervix)) OR (TS=(Cervix Cancer))) |  |
| #13 | ((TS=(Endometrium Neoplasm*)) OR (TS=(Endometrial Neoplasm*)) OR (TS=(Endometrial Carcinoma*)) OR (TS=(Endometrium Cancinoma*)) OR (TS=(Endometrial Cancer*)) OR (TS=(Endometrium Cancer*))) |  |
| #14 | ((TS=(Ovarian Neoplasm*)) OR (TS=(Ovary Neoplasm*)) OR (TS=(Ovary Cancer*)) OR (TS=(Ovarian Cancer*))) |  |
| #15 | #1 AND #6 AND #7 AND #8 AND DOP=(2005-01-01/2022-12-05) and Humans (MeSH Headings) and English (Languages) | 15 |
| #16 | #1 AND #6 AND #7 AND #9 AND DOP=(2005-01-01/2022-12-05) and Humans (MeSH Headings) and English (Languages) | 40 |
| #17 | #1 AND #6 AND #7 AND #10 AND DOP=(2005-01-01/2022-12-05) and Humans (MeSH Headings) and English (Languages) | 7 |
| #18 | #1 AND #6 AND #7 AND #11 AND DOP=(2005-01-01/2022-12-05) and Humans (MeSH Headings) and English (Languages) | 90 |
| #19 | #1 AND #6 AND #7 AND #12 AND DOP=(2005-01-01/2022-12-05) and Humans (MeSH Headings) and English (Languages) | 101 |
| #20 | #1 AND #6 AND #7 AND #13 AND DOP=(2005-01-01/2022-12-05) and Humans (MeSH Headings) and English (Languages) | 12 |
| #21 | #1 AND #6 AND #7 AND #14 AND DOP=(2005-01-01/2022-12-05) and Humans (MeSH Headings) and English (Languages) | 27 |
| #22 | #2 AND #6 AND #7 AND #8 AND DOP=(2005-01-01/2022-12-05) and Humans (MeSH Headings) and English (Languages) | 15 |
| #23 | #2 AND #6 AND #7 AND #9 AND DOP=(2005-01-01/2022-12-05) and Humans (MeSH Headings) and English (Languages) | 7 |
| #24 | #2 AND #6 AND #7 AND #10 AND DOP=(2005-01-01/2022-12-05) and Humans (MeSH Headings) and English (Languages) | 34 |
| #25 | #2 AND #6 AND #7 AND #11 AND DOP=(2005-01-01/2022-12-05) and Humans (MeSH Headings) and English (Languages) | 609 |
| #26 | #2 AND #6 AND #7 AND #12 AND DOP=(2005-01-01/2022-12-05) and Humans (MeSH Headings) and English (Languages) | 250 |
| #27 | #2 AND #6 AND #7 AND #13 AND DOP=(2005-01-01/2022-12-05) and Humans (MeSH Headings) and English (Languages) | 14 |
| #28 | #2 AND #6 AND #7 AND #14 AND DOP=(2005-01-01/2022-12-05) and Humans (MeSH Headings) and English (Languages) | 9 |
| #29 | #3 AND #6 AND #7 AND (#8 OR #12 OR #13 OR #14) AND DOP=(2005-01-01/2022-12-05) and Humans (MeSH Headings) and English (Languages) | 9 |
| #30 | #4 AND #6 AND #7 AND #13 AND DOP=(2005-01-01/2022-12-05) and Humans (MeSH Headings) and English (Languages) | 42 |
| #31 | #5 AND #6 AND #7 AND #14 AND DOP=(2005-01-01/2022-12-05) and Humans (MeSH Headings) and English (Languages) | 142 |

**1.4 Embase**

| ID | Search | Hits |
| --- | --- | --- |
| #1 | microbiome:ti,ab,kw OR microbiota:ti,ab,kw OR ecosystem:ti,ab,kw OR bacteria:ti,ab,kw OR flora:ti,ab,kw OR microflora:ti,ab,kw OR dysbiosis:ti,ab,kw | 717877 |
| #2 | 16s:ti,ab,kw OR sequencing:ti,ab,kw OR shotgun:ti,ab,kw OR metagenome:ti,ab,kw OR ngs:ti,ab,kw OR illumina:ti,ab,kw OR pyrosequencing:ti,ab,kw | 654356 |
| #3 | gut:ti,ab,kw OR gastrointestinal:ti,ab,kw OR intestinal:ti,ab,kw OR feacal:ti,ab,kw OR fecal:ti,ab,kw OR stool:ti,ab,kw OR feces:ti,ab,kw | 982988 |
| #4 | 'genital tract':ti,ab,kw OR vaginal:ti,ab,kw OR 'cervix mucus':ti,ab,kw OR 'cervical mucus':ti,ab,kw OR 'cervical swab':ti,ab,kw | 173121 |
| #5 | 'peritoneal fluid':ti,ab,kw OR 'ascitic fluid':ti,ab,kw OR 'peritoneal effusion':ti,ab,kw | 14197 |
| #6 | 'endometrial tissue':ti,ab,kw OR 'endometrium biopsy':ti,ab,kw OR 'endometrial biopsy':ti,ab,kw OR 'endometrial sample':ti,ab,kw OR 'endometrium tissue':ti,ab,kw OR 'endometrium sample':ti,ab,kw | 9879 |
| #7 | 'ovarian tissue':ti,ab,kw OR 'ovarian surface tissue':ti,ab,kw OR 'ovarian cancer tissue':ti,ab,kw OR 'ovarian biopsy':ti,ab,kw OR 'ovarian sample':ti,ab,kw | 8785 |
| #8 | microbiome:ti,ab,kw OR microbiota:ti,ab,kw OR ecosystem:ti,ab,kw OR flora:ti,ab,kw OR microflora:ti,ab,kw OR dysbiosis:ti,ab,kw | 242428 |
| #9 | 'vaginitis'/exp OR 'bacterial vaginosis':ti,ab,kw OR 'bacterial vaginitides':ti,ab,kw OR 'bacterial vaginoses':ti,ab,kw OR 'bacterial vaginitis':ti,ab,kw | 19460 |
| #10 | 'endometriosis'/exp OR 'adenomyosis externa':ti,ab,kw OR 'endometriosis externa':ti,ab,kw OR endometrioma:ti,ab,kw OR endometrioses:ti,ab,kw | 47633 |
| #11 | 'ovary polycystic disease'/exp OR 'polycystic ovary syndrome':ti,ab,kw OR 'polycystic ovarian syndrome':ti,ab,kw OR 'stein leventhal syndrome':ti,ab,kw | 37351 |
| #12 | uterine cervix carcinoma'/exp OR 'uterine cervix cancer'/exp OR 'cervical intraepithelial neoplasia':ti,ab,kw OR 'cervical intraepithelial neoplasm':ti,ab,kw OR 'squamous intraepithelial lesion':ti,ab,kw OR 'cervical cancer':ti,ab,kw OR 'cervix cancer':ti,ab,kw OR 'uterine cervical neoplasm':ti,ab,kw | 136850 |
| #13 | ovary cancer'/exp OR 'ovarian neoplasms':ti,ab,kw OR 'ovarian cancer':ti,ab,kw | 159283 |
| #14 | endometrium cancer'/exp OR 'endometrial carcinoma':ti,ab,kw OR 'endometrium carcinoma':ti,ab,kw OR 'endometrial cancer':ti,ab,kw | 63935 |
| #15 | uterus myoma'/exp OR 'adenomyosis'/exp OR 'endometritis'/exp OR leiomyoma:ti,ab,kw OR fibromyoma:ti,ab,kw OR 'fibroid uterus':ti,ab,kw OR 'uterine fibroma':ti,ab,kw OR 'uterine fibroid':ti,ab,kw OR adenomyoses:ti,ab,kw OR endomyometritis:ti,ab,kw | 43965 |
| #16 | #2 AND #3 AND #8 AND #9 AND [english]/lim AND [humans]/lim AND [embase]/lim AND [01-01-2005]/sd NOT [06-12-2022]/sd | 29 |
| #17 | #1 AND #2 AND #3 AND #10 AND [english]/lim AND [humans]/lim AND [embase]/lim AND [01-01-2005]/sd NOT [06-12-2022]/sd | 13 |
| #18 | #1 AND #2 AND #3 AND #11 AND [english]/lim AND [humans]/lim AND [embase]/lim AND [01-01-2005]/sd NOT [06-12-2022]/sd | 51 |
| #19 | #1 AND #2 AND #3 AND #12 AND [english]/lim AND [humans]/lim AND [embase]/lim AND [01-01-2005]/sd NOT [06-12-2022]/sd | 30 |
| #20 | #1 AND #2 AND #3 AND #13 AND [english]/lim AND [humans]/lim AND [embase]/lim AND [01-01-2005]/sd NOT [06-12-2022]/sd | 18 |
| #21 | #1 AND #2 AND #3 AND #14 AND [english]/lim AND [humans]/lim AND [embase]/lim AND [01-01-2005]/sd NOT [06-12-2022]/sd | 16 |
| #22 | #1 AND #2 AND #3 AND #15 AND [english]/lim AND [humans]/lim AND [embase]/lim AND [01-01-2005]/sd NOT [06-12-2022]/sd | 5 |
| #23 | #2 AND #4 AND #8 AND #9 AND [english]/lim AND [humans]/lim AND [embase]/lim AND [01-01-2005]/sd NOT [06-12-2022]/sd | 416 |
| #24 | #1 AND #2 AND #4 AND #10 AND [english]/lim AND [humans]/lim AND [embase]/lim AND [01-01-2005]/sd NOT [06-12-2022]/sd | 25 |
| #25 | #1 AND #2 AND #4 AND #11 AND [english]/lim AND [humans]/lim AND [embase]/lim AND [01-01-2005]/sd NOT [06-12-2022]/sd | 11 |
| #26 | #1 AND #2 AND #4 AND #12 AND [english]/lim AND [humans]/lim AND [embase]/lim AND [01-01-2005]/sd NOT [06-12-2022]/sd | 89 |
| #27 | #1 AND #2 AND #4 AND #13 AND [english]/lim AND [humans]/lim AND [embase]/lim AND [01-01-2005]/sd NOT [06-12-2022]/sd | 13 |
| #28 | #1 AND #2 AND #4 AND #14 AND [english]/lim AND [humans]/lim AND [embase]/lim AND [01-01-2005]/sd NOT [06-12-2022]/sd | 12 |
| #29 | #1 AND #2 AND #4 AND #15 AND [english]/lim AND [humans]/lim AND [embase]/lim AND [01-01-2005]/sd NOT [06-12-2022]/sd | 30 |
| #30 | #1 AND #2 AND #5 AND #10 AND [english]/lim AND [humans]/lim AND [embase]/lim AND [01-01-2005]/sd NOT [06-12-2022]/sd | 6 |
| #31 | #1 AND #2 AND #5 AND #12 AND [english]/lim AND [humans]/lim AND [embase]/lim AND [01-01-2005]/sd NOT [06-12-2022]/sd | 0 |
| #32 | #1 AND #2 AND #5 AND #13 AND [english]/lim AND [humans]/lim AND [embase]/lim AND [01-01-2005]/sd NOT [06-12-2022]/sd | 1 |
| #33 | #1 AND #2 AND #5 AND #14 AND [english]/lim AND [humans]/lim AND [embase]/lim AND [01-01-2005]/sd NOT [06-12-2022]/sd | 0 |
| #34 | #1 AND #2 AND #6 AND #14 AND [english]/lim AND [humans]/lim AND [embase]/lim AND [01-01-2005]/sd NOT [06-12-2022]/sd | 3 |
| #35 | #1 AND #2 AND #7 AND #13 AND [english]/lim AND [humans]/lim AND [embase]/lim AND [01-01-2005]/sd NOT [06-12-2022]/sd | 2 |

Table S1. Detailed characteristics of the included studies

| Study ID | Country | Disease | Participants | Age | BMI |
| --- | --- | --- | --- | --- | --- |
| Oakley 2008 ^[1]^ | USA | BV | 41 |  |  |
| Ling 2010 ^[2]^ | China | BV | 50 BV positive 50 Healthy women | P: 33.3±9.1 C: 32.0±8.1 |  |
| Srinivasan 2012 ^[3]^ | USA | BV | 98 BV and 121 controls by Amsel’s criteria 117 BV and 103 controls by Nugent Score | 29.2 |  |
| Shipitsyna 2013 ^[4]^ | Russia | BV | 79 controls  73 BV  11 intermediate (Lactobacillary grade II flora) |  |  |
| Dols 2016 ^[5]^ | Holland | BV | 20 BV 20 Control | P:22.5 (21~27) C:23 (22~25) |  |
| Zozaya 2016 ^[6]^ | USA | BV | 65 BV 31 controls | P: 27.3 ± 6.6 C: 28.4 ± 7.7 |  |
| Chen 2018 ^[7]^ | China | BV | 19: A-N- 11: A+N+ | A-N-: 41±10.42 A+N+: 31±6.49 |  |
| Ceccarani 2019 ^[8]^ | Italy | BV | 20:BV 20: CT 18: VVC 21: HC | BV:29.3±7.7 CT:24.3±3.4 VVC:27.8±7.2 HC:26.1±6.3 | BV:22.9±2.4 CT:23.6±1.8 VVC:23.0±2.0 HC:23.8±2.4 |
| Lynch 2019 ^[9]^ | Canada | BV | 57: Symptomatic 36: Asymptomatic 35 BV (qPCR) 33 Negative (qPCR) |  |  |
| Tao 2019 ^[10]^ | China | BV | 10: BV 10: AV 21: HC | 18~50 |  |
| Oerlemans 2020 ^[11]^ | Belgium | BV | 20: BV 20: AV 18: Control | 18~51 |  |
| Pramanick 2021 ^[12]^ | India | BV | 20: BV 19: Control | BV:33.26±5.42 C:30.79±7.40 |  |
| Deng 2022 ^[13]^ | China | BV | 37: BV 33: BV intermediate 39: Healthy control | BV:38.9±8.03 intermediate:37.9±6.03 HC:39.6±7.15 |  |
| Khan 2016 ^[14]^ | Japan | EM | GnRHa(−)  16 control with uterine myoma,  16 endometriosis | P:35.7±8.3 C:33.6±8.9 |  |
| Ata 2019 ^[15]^ | Turkey | EM | 14 Caucasian women with endometriosis, 14 Caucasian healthy controls | P:28.5 (26–31.3)  C:27.5 (25.8–30) | P:23 (21–24.3)  C:21 (20.1–24.2) |
| Akiyama 2019 ^[16]^ | Japan | EM | 30 r-ASRM stage III-IV endometriosis 39 control (fibroids or benign ovarian tumor) | P:33.9±5.7 C:32.5±6.0 | P:20.58±2.89  C:21.33±3.27 |
| Wei 2020 ^[17]^ | China | EM | 16 I-II stage 20 III-IV stage 14 control (7 ovarian teratoma, 4 serous cystadenoma, 3 uterine fibroids) | 31.47 |  |
| Hernandes 2020 ^[18]^ | Brazil | EM | 10 with deep endometriosis  11 control (laparoscopic surgery for benign gynecologic diseases or elective tubal ligation) |  |  |
| Chen 2020a ^[19]^ | China | EM | 20 endometriosis (ovarian endometriosis, DIE, peritoneal type and other special types)  36 control group | 36.07±5.57 | 21.88±2.93 |
| Huang 2021 ^[20]^ | China | EM | 21 endometriosis  20 control | P: 38.3 ± 7.88 C: 34.0 ± 10.8 | P: 21.5 ± 2.79 C: 24.3 ± 8.16 |
| Svensson 2021 ^[21]^ | Sweden | EM | 66 endometriosis 198 controls (41 recent therapy) | P: 37.8(32.8-43.3) C: 37.0(32.0-44.0) | P: 25.0 (22.0–28.0) C: 24.7 (22.1–27.5) |
| Shan 2021 ^[22]^ | China | EM | 12 with stage 3/4 EM  12 healthy volunteers | P: 32 ± 2 C: 32 ± 3 |  |
| Le 2021 ^[23]^ | USA | EM | 9 non-diseased patients 20 pathologically confirmed endometriosis (5 I, 3 II, 4 III, 8 IV) | P: 32.5 ± 1.1 C: 32.6 ± 2.0 | P:26.5 ± 1.5 C: 28.1 ± 2.4 |
| Wessels 2021 ^[24]^ | Canada | EM | 14 endometriosis (1 II, 1 III, 12 IV) 10 controls (other benign gynecological conditions) | P: 33.8±5.8 C: 35.1±3.3 |  |
| Lee 2021 ^[25]^ | Korea | EM | 45 women with histological evidence of endometriosis 45 controls (31 uterine leiomyoma, 14 benign ovarian cyst) | P: 36.2 ± 1.3 C: 39.4 ± 1.1 | P: 21.0 ± 0.5 C: 22.2 ± 0.5 |
| Yuan 2022 ^[26]^ | China | EM | 2 stage I, 5 stage II  15 stage III, 14 stage IV | P: 35.28 ±7.24 C: 33.32 ±8.04 | P: 20.99 ± 2.11 C: 21.43 ± 2.03 |
| Kunaseth 2022 ^[27]^ | Thailand | AM | 40: Adenomyosis 40: Control | P:42.7±5.7 C: 42.4±5.0 | P:24.2±4.2 C: 24.8±5.3 |
| Lindheim 2017 ^[28]^ | Austria | PCOS | 20 healthy controls (19 provided a stool sample) 24 PCOS patients | P: 27 (5.9)  C: 32 (12) | P: 24.9 (11.75)  C: 22.3 (4.10) |
| Liu 2017 ^[29]^ | China | PCOS | 12 non-obese PCOS (PN) 21 obese PCOS (PO) 9 non-obese controls (CN) 6 obese controls (CO) BMI ≥ 25, WHO2020 | PN: 25.5 ± 4.3 PO: 29.3 ± 6.5 CN: 32.2 ± 5.9 CO: 33 ± 5.4 | PN: 21.6 ± 2.2 PO: 30 ± 3.6 CN: 21.9 ± 2.2 CO: 27.5 ± 3.3 |
| Torres 2018 ^[30]^ | USA/  Poland | PCOS | 48 healthy women 42 women with polycystic ovarian morphology (PCOM) 73 PCOS using Rotterdam criteria | P: 27.4 ± 4.9 C: 29.4 ± 4.9 | P: 25.6 ± 6.5 C: 23.7 ± 4.1 |
| Qi 2019 ^[31]^ | China | PCOS | 50 PCOS, 43 controls | P: 29.9±0.45 C: 29.6±0.56 | P: 24.7±0.51 C: 23.7±0.59 |
| Zeng 2018 ^[32]^ | China | PCOS | 9 PCOS with insulin resistance 8 PCOS alone 8 healthy controls | IR-PCOS: 25.11 ± 4.28 NIR-PCOS: 26.13 ± 7.06 C: 26.38 ± 3.85 | IR-PCOS: 22.63 ± 2.40 NIR-PCOS: 22.58 ± 2.37 C: 20.82 ± 2.28 |
| Hong 2020 ^[33]^ | China | PCOS | 39 PCOS, 40 controls | P: 26.26 ± 4.45 C: 27.33 ± 4.06 |  |
| Zhou 2020a ^[34]^ | China | PCOS | 18 obese PCOS 15 obese controls obese BMI ≥ 28 | PO: 26 ± 4 CO: 24 ± 1 | PO: 29.78 ± 1.40 CO: 29.72 ± 1.48 |
| Eyupoglu 2020 ^[35]^ | Turkey | PCOS | 17 overweight (BMI ≥ 25) obese (BMI ≥ 30) PCOS 15 age- and BMI- matched healthy controls | PO: 20 (19–22)  CO: 22 (18–27) | PO: 29.6 (27.5–32.2) CO: 31.5 (28–35.3) |
| Liang 2020 ^[36]^ | China | PCOS | 8 obese women with PCOS (BMI ≥ 25) (PO group) 10 normal-weight PCOS women (BMI < 30) (PN group) 9 healthy normal-weight women (control) (C group) | PO: 27.1±3.5 PN: 25.7±3.5 C: 27.9±3.6 | PO: 29.5±4.8 PN: 20.7±1.9 C: 20.9±2.6 |
| Chu 2020 ^[37]^ | China | PCOS | 7 nonoverweight PCOS 7 overweight PCOS 7 nonoverweight controls 7 overweight controls overweight BMI ≥ 24 | PN: 27.14±4.56 PO: 29.14±2.87 CN: 30.29±3.90 CO: 28.57±2.72 | PN: 20.98±0.89 PO: 27.93±4.23 CN: 20.58±4.46 CO: 27.06±3.92 |
| Zhou 2020b ^[38]^ | China | PCOS | 30 non-obese PCOS (NG) (BMI<24) 30 non-obese controls (NC) (BMI<24) 30 obese PCOS (OG) (BMI ≥28) 11 obese controls (OC) (BMI ≥28) | NG: 25.1 ± 4.27 NC: 22.1 ± 1.64 OG: 26.9 ± 4.86 OC: 25.3 ± 1.61 |  |
| Tu 2020 ^[39]^ | China | PCOS | 47 PCOS, 50 controls |  | P: 24.4 ± 3.8 C: 21.8 ± 2.7 |
| Lu 2021a ^[40]^ | China | PCOS | 42 PCOS  24 healthy volunteers who received assisted reproductive technology due to male infertility | P: 28.071±3.872 C: 29.458±4.549 | P: 25.758±4.425 C: 21.790±2.273 |
| Lüll 2021 ^[41]^ | Finland | PCOS | 102 PCOS women  201 non-PCOS control women |  |  |
| Liang 2021 ^[42]^ | China | PCOS | 10 lean PCOS, PL 10 overweight PCOS, PO 10 lean control, CL 10 overweight control, CO overweight BMI ≥ 23  (Previous study and the WHO recommendation for Asian populations) | PL: 24.13 ± 2.45 CL: 25.08 ± 3.59 PO: 28.94 ± 6.13 CO: 30.12 ± 5.20 | PL: 20.46 ± 1.58 CL: 20.43 ± 1.19 PO: 27.34 ± 3.51 CO: 26.05 ± 3.13 |
| Mammadova 2021 ^[43]^ | Turkey | PCOS | 24 lean PCOS 22 healthy lean controls (BMI≤25) | PN: 19.5 (19.0-22.5)  CN: 23.0 (22.0-24.3) | PN: 22.9 ± 3.2 CN: 22.5 ± 2.5 |
| Insenser 2018 ^[44]^ | Spain | PCOS | 15 PCOS (7 nonobese, 8 obese) 16 Controls women (8 nonobese, 8 obese) 15 Controls men obese BMI≥30 according to their BMI | PO: 29.9 ± 5.1 PN: 23.0 ± 7.9 CO: 27.3 ± 6.5 CN: 27.3 ± 4.0 | PO: 37.0 ± 4.8 PN: 24.4 ± 2.8 CO: 35.9 ± 4.2 CN: 23.4 ± 1.9 |
| He 2021 ^[45]^ | China | PCOS | 14 PCOS patients with insulin resistant (PCOS-IR) 12 PCOS alone (PCOS-NIR) 10 healthy control women (HC) (ALL BMI<24) | IR: 26.71 ± 2.43 NIR: 26.4 ± 3.41 HC: 28.25 ± 1.22 | IR: 21.91 ± 1.4 NIR: 21.32 ± 1.22 HC: 21.28 ± 1.34 |
| Dong 2021 ^[46]^ | China | PCOS | 14 not overweight PCOS (NOW) (BMI<24) 31 overweightobese PCOS (OW) (BMI ≥24) 25 not overweight control (NOW) (BMI<24) 12 overweightobese control (OW) (BMI ≥24) | P: 30(27-34) C: 31(28-33.5) | P: 26.95(22.89-31.25) C: 22.58(20.14-25.09) |
| Yang 2021 ^[47]^ | China | PCOS | 56 PCOS, 31 controls | P: 24.00 (22.00, 27.00) C: 26.00 (24.00, 27.00) | P: 21.07 (18.75, 24.41) C: 19.81 (18.76, 21.16) |
| Zhu 2021 ^[48]^ | China | PCOS | 54 premenopausal PCOS women 33 premenopausal age controls |  |  |
| Chen 2021 ^[49]^ | China | PCOS | 38 HC (normal BMI)  48 PCOS-LB (BMI＜24)  50 PCOS-HB (BMI＞24) | C: 29.26 ± 4.18 PCOS-LB: 29.48 ± 3.39 PCOS-HB: 29.64 ± 4.06 | C: 20.19 ± 1.6 PCOS-LB: 20.57 ± 2.12 PCOS-HB: 28.18 ± 2.82 |
| Hassan 2022 ^[50]^ | India | PCOS | 19 PCOS 20 controls | P: 23.9 ± 6.9 C: 21.1 ± 2.5 | P: 25.4 ± 3.3 C: 23.2 ± 3.2 |
| Li 2022 ^[51]^ | China | PCOS | 31 PCOS 27 controls | P: 24.35 ± 4.48 C: 27.0 ± 4.90 | P: 24.19 ± 3.87 C: 23.89 ± 3.95 |
| Yang 2022 ^[52]^ | China | PCOS | 32 PCOS 18 Control | P:29.34±2.88 C:29.89±3.36 | P:23.46±3.67 C:20.44±2.20 |
| Yin 2022 ^[53]^ | China | PCOS | 21 Healthy-LB (BMI < 24) 22 PCOS-LB (BMI < 24) 20 Healthy-HB (BMI ≥ 24) 25 PCOS-HB (BMI ≥ 24) | PCOS-LB: 28.96 ± 3.87 PCOS-HB: 29.04 ± 3.89 Healthy-LB: 28.53 ± 3.19 Healthy-HB: 29.9 ± 2.98 | PCOS-LB: 20.68 ± 2.17 PCOS-HB: 28.19 ± 2.82 Healthy-LB: 20.01 ± 1.83 Healthy-HB: 29.03 ± 3.06 |
| Oh 2015 ^[54]^ | Korea | CIN | 55 CIN 1 15 CIN 2 or 3 25 control women with normal cytology 25 ASCUS | P: 44.7 C: 42.5 |  |
| Zhang 2018 ^[55]^ | China | CIN | 126 CIN 1− (normal cytology and CIN 1)  40 CIN 2+ (CIN 2 and CIN 3) |  |  |
| Klein 2019 ^[56]^ | Tanzania | CIN | 45:HSIL 67:LSIL 22:NILM | Mean 37 |  |
| Wu 2020 ^[57]^ | China | CIN | 16:HSIL 22:LSIL 31:NILM | HSIL:41±9.11 LSIL:38±9.84 NILM:37±8.34 |  |
| Lee 2020 ^[58]^ | Korea | CIN | 24:CIN1- (HC to CIN 1) 42:CIN2+ (CIN 2+ to CCA) | CIN1-:49.2±7.3 CIN2+:42.7±13.2 |  |
| Chao 2021 ^[59]^ | China | CIN | 83:CIN2+ (HPV+ and HSIL) 86:HPV+ (without cervical neoplasia) 103:HPV- | CIN2+:38.34±10.18 HPV infected:39.00±9.28 HPV negative:39.35±9.43 |  |
| Nieves-Ramirez 2021 ^[60]^ | Mexico | CIN | 121:SIL positive (90 LSIL and 31 HSIL) 107:SIL negative (35 HPV+ and 72 HPV-) | CIN:37.26±10.87 Normal:42.83±7.92 |  |
| Lin 2022 ^[61]^ | China | CIN | 60:CIN (41 CIN1, 6 CIN2, 13 CIN3) 60:HC (5160 HPV+) | CIN:40.00(30.75~47.25) HC:38.00(29.75~49.00) | CIN:21.75±2.80 HC:22.55±3.95 |
| Xia 2022 ^[62]^ | China | CIN | 34:LSIL 58:HPV(+) 43:HPV(-) | LSIL:32.06±6.48 HPV(+):31.36±5.86 HPV(-):33.65±5.25 | p＝0.993 |
| Zhang 2022 ^[63]^ | China | CIN | 113:HC 159:HPV+ 84:CIN (47 CIN1, 24 CIN2, 13 CIN3) | 20~69 |  |
| Mitra 2015 ^[64]^ | UK | CIN-CCA | 20 control 52 LSIL 92 HSIL 5 invasive cervical cancer(ICC) | C:31±5.19 LSIL: 32±5.66 HSIL: 30±4.65 ICC: 34±5.29 |  |
| Audirac-Chalifour 2016 ^[65]^ | Mexico | CIN-CCA | 20 non-cervical lesions (NCL: 10 HPV-, 10 HPV+) 4 SILs (HPV+)  8 CC (HPV+) | NCL: 34(8) SIL: 40(14) CC: 43(11) |  |
| Laniewski 2018 ^[66]^ | USA | CIN-CCA | 20 Ctrl HPV− 31 Ctrl HPV+ 12 low-grade dysplasia 27 high-grade dysplasia 10 invasive cervical carcinoma | 39.55 (7.35) 37.64 (9.38) 35.08 (7.24) 38.29 (8.46) 38.90 (9.09) |  |
| Wang 2019 ^[67]^ | China | CCA | 8 cervical cancer (CCa) 5 age-matched healthy female controls (HCs) | 59.5 |  |
| Kwon 2019 ^[68]^ | Korea | CCA | 18 normal 17 CIN 2/3 12 cervical cancer | C: 45.6 (7.72) CIN: 41.1 (6.98) CC: 55.9 (10) | C: 21.3 (2.66) CIN: 21.3 (2.56) CC: 22.9 (4.03) |
| Sims 2019 ^[69]^ | USA | CCA | 42 cervical cancer 46 healthy female controls | P: 48.9 (10.4) C: 48.9 (13.7) | P: 29.0 (6.6) C: 29.6 (8.3) |
| Tango 2020 ^[70]^ | Korea | CIN- CCA | 50 control 42 CIN23-CC | P: 45.7 (11.7) C: 45.1 (11.6) | P: 22.1 (2.9) C: 21.9 (2.8) |
| Chen 2020b ^[71]^ | China | CIN- CCA | 68 HPV(-) Healthy Women 78 HPV(+) 51 LSIL 23 HSIL 9 invasive cervical cancer | C: 43.00±8.69 HPV: 47.78±9.63 LSIL: 46.00±10.19 HSIL: 43.70±10.74 CC: 56.11±9.02 | C: 22.94±2.74 HPV: 22.76±2.97 LSIL: 22.44±2.94 HSIL: 22.85±2.70 CC: 23.99±0.68 |
| So 2020 ^[72]^ | Korea | CIN-CCA | 10:CCA 10:CIN2+ 10:CIN1 10:Normal | 20~50 |  |
| Xie 2020 ^[73]^ | China | CIN-CCA | 23:CCA 22:CIN 27:Control | CCA:34.16±1.914 CIN:35.53±2.951 Control: 34.04±3.142 |  |
| Wu 2021 ^[74]^ | China | CIN-CCA | 13:CCA 31:HSIL 10:LSIL 12:NH (HPV+) 28:NN (HPV-) | CCA:42.8±8.0 HSIL:40±7.3 LSIL:37.2±6.3 NH:37.1±9.5 NN:37.5±7.2 |  |
| Zhai 2021 ^[75]^ | China | CIN-CCA | 38:CCA 40:HSIL 32:LSIL 29:hrHPV(+) 29:HC | CCA:42.43±5.31 HSIL:40.64±5.57 LSIL:40.63±4.55 hrHPV(+):42.17±5.18 HC:40.08±4.83 | CCA:22.88±1.96 HSIL:22.37±1.62 LSIL:22.32±2.93 hrHPV(+):22.00±2.16 HC:23.20±3.47 |
| Zhang 2021 ^[76]^ | China | CCA | 10:CCA 38:HPV1618(+) 32:Other hrHPV(+) 20:Normal | CCA:38.80±3.08 HPV1618(+):35.73±6.89 Other hrHPV(+):36.75±6.15 Normal:38.35±3.72 |  |
| Kang 2020 ^[77]^ | Korea | CCA | 17:CCA 29:HC | CCA:43.9±9.9 HC:36.1±9.9 |  |
| Mao 2022 ^[78]^ | China | UF | 43:Uterine fibroid 42:Control | P: 43(24~52) C: 35 (23~54) | P: 23.34±3.22 C: 22.48±3.53 |
| Walther-António 2016 ^[79]^ | USA | EC | 10 control (benign gynecologic condition) 4 endometrial hyperplasia 17 endometrial cancer | P: 64 (58–71) C: 44.5 (42.5–52.5) | P: 32.1 (26.8–40.2) C: 26.6 (23.8–34.1) |
| Lu 2021b ^[80]^ | China | EC | 25 EC, 25 benign uterine lesions |  |  |
| Gressel 2021 ^[81]^ | USA | EC | 35 postmenopausal women  (10 controls-Benign 14 Endometrioid Endometrial Adenocarcinoma 11 Uterine Serous Cancers) | C: 59.0 ± 7.2 EAC: 61.4 ± 9.4 USC: 70.5 ± 6.6 | C: 29.2 (26.4, 36.3) EAC: 38.0 (33.1, 42.5) USC: 32.4 (24.7, 36.1) |
| Li 2021 ^[82]^ | China | EC | 30 EC, 10 controls | P: 56.4 ± 7.89 C: 53.1 ± 6.67 | P: 22.70 ± 1.66 C: 22.89 ± 2.15 |
| Hawkins 2022 ^[83]^ | USA | EC | 23 Black EC, 72 White EC, 16 benign indication |  |  |
| Nené 2019 ^[84]^ | UK | OC | 176 epithelial ovarian cancer 184 control (115 healthy controls) 69 controls with benign gynaecological conditions |  |  |
| Zhou 2019 ^[85]^ | China | OC | 25 ovarian cancer tissues (C group)  25 normal distal fallopian tube tissues (N group) | P: 54.5 ± 7.3 C: 48.2 ± 7.7 |  |
| Wang 2020 ^[86]^ | China | OC | 6 OC 10 controls (3 uterine myoma, 7 uterine adenomyosis) | P: 57.3 C: 51.6 |  |
| Morikawa 2022 ^[87]^ | Japan | OC | 39 OC, 20 healthy controls, 3 with ovarian benign tumors | MEDIAN P: 56 C: 47 |  |

BMI, body mass index; P, patient; C, control; HC, healthy control; BV, bacterial vaginosis; PCOS, polycystic ovary syndrome; EM, endometriosis; AM, adenomyosis; CIN, cervical intraepithelial neoplasia; CCA, cervical cancer; UF, uterine fibroid; EC, endometrial cancer; OC, ovarian cancer. The data was shown as Mean ± SD or Median (IQR).

**References:**

1. Oakley BB, Fiedler TL, Marrazzo JM, Fredricks DN. Diversity of Human Vaginal Bacterial Communities and Associations with Clinically Defined Bacterial Vaginosis. *Appl Environ Microbiol*. 2008;74(15):4898–4909. doi:10.1128/AEM.02884-07

2. Ling Z, Kong J, Liu F, Zhu H, Chen X, Wang Y, Li L, Nelson KE, Xia Y, Xiang C. Molecular analysis of the diversity of vaginal microbiota associated with bacterial vaginosis. *BMC Genomics*. 2010;11(1):488. doi:10.1186/1471-2164-11-488

3. Srinivasan S, Hoffman NG, Morgan MT, Matsen FA, Fiedler TL, Hall RW, Ross FJ, McCoy CO, Bumgarner R, Marrazzo JM, Fredricks DN. Bacterial Communities in Women with Bacterial Vaginosis: High Resolution Phylogenetic Analyses Reveal Relationships of Microbiota to Clinical Criteria. *PLoS ONE*. 2012;7(6):e37818. doi:10.1371/journal.pone.0037818

4. Shipitsyna E, Roos A, Datcu R, Hallén A, Fredlund H, Jensen JS, Engstrand L, Unemo M. Composition of the Vaginal Microbiota in Women of Reproductive Age – Sensitive and Specific Molecular Diagnosis of Bacterial Vaginosis Is Possible? *PLoS ONE*. 2013;8(4):e60670. doi:10.1371/journal.pone.0060670

5. Dols JAM, Molenaar D, van der Helm JJ, Caspers MPM, de Kat Angelino-Bart A, Schuren FHJ, Speksnijder AGCL, Westerhoff HV, Richardus JH, Boon ME, et al. Molecular assessment of bacterial vaginosis by Lactobacillus abundance and species diversity. *BMC Infect Dis*. 2016;16(1):180. doi:10.1186/s12879-016-1513-3

6. Zozaya M, Ferris MJ, Siren JD, Lillis R, Myers L, Nsuami MJ, Eren AM, Brown J, Taylor CM, Martin DH. Bacterial communities in penile skin, male urethra, and vaginas of heterosexual couples with and without bacterial vaginosis. *Microbiome*. 2016;4(1):16. doi:10.1186/s40168-016-0161-6

7. Chen H-M, Chang T-H, Lin F-M, Liang C, Chiu C-M, Yang T-L, Yang T, Huang C-Y, Cheng Y-N, Chang Y-A, et al. Vaginal microbiome variances in sample groups categorized by clinical criteria of bacterial vaginosis. *BMC Genomics*. 2018;19(S10):876. doi:10.1186/s12864-018-5284-7

8. Ceccarani C, Foschi C, Parolin C, D’Antuono A, Gaspari V, Consolandi C, Laghi L, Camboni T, Vitali B, Severgnini M, Marangoni A. Diversity of vaginal microbiome and metabolome during genital infections. *Sci Rep*. 2019;9(1):14095. doi:10.1038/s41598-019-50410-x

9. Lynch T, Peirano G, Lloyd T, Read R, Carter J, Chu A, Shaman JA, Jarvis JP, Diamond E, Ijaz UZ, Church D. Molecular Diagnosis of Vaginitis: Comparing Quantitative PCR and Microbiome Profiling Approaches to Current Microscopy Scoring. *J Clin Microbiol*. 2019;57(9):e00300-19. doi:10.1128/JCM.00300-19

10. Tao Z, Zhang L, Zhang Q, Lv T, Chen R, Wang L, Huang Z, Hu L, Liao Q. The Pathogenesis Of Streptococcus anginosus In Aerobic Vaginitis. *IDR*. 2019;Volume 12:3745–3754. doi:10.2147/IDR.S227883

11. Oerlemans EFM, Wuyts S, Bellen G, Wittouck S, De Boeck I, Ruban K, Allonsius CN, van den Broek MFL, Donders GGG, Lebeer S. The Dwindling Microbiota of Aerobic Vaginitis, an Inflammatory State Enriched in Pathobionts with Limited TLR Stimulation. *Diagnostics*. 2020;10(11):879. doi:10.3390/diagnostics10110879

12. Pramanick R, Nathani N, Warke H, Mayadeo N, Aranha C. Vaginal Dysbiotic Microbiome in Women With No Symptoms of Genital Infections. *Front Cell Infect Microbiol*. 2022;11:760459. doi:10.3389/fcimb.2021.760459

13. Deng T, Shang A, Zheng Y, Zhang L, Sun H, Wang W. Log ( *Lactobacillus crispatus* / *Gardnerella vaginalis* ): a new indicator of diagnosing bacterial vaginosis. *Bioengineered*. 2022;13(2):2981–2991. doi:10.1080/21655979.2022.2027059

14. Khan KN, Fujishita A, Masumoto H, Muto H, Kitajima M, Masuzaki H, Kitawaki J. Molecular detection of intrauterine microbial colonization in women with endometriosis. *European Journal of Obstetrics & Gynecology and Reproductive Biology*. 2016;199:69–75. doi:10.1016/j.ejogrb.2016.01.040

15. Ata B, Yildiz S, Turkgeldi E, Brocal VP, Dinleyici EC, Moya A, Urman B. The Endobiota Study: Comparison of Vaginal, Cervical and Gut Microbiota Between Women with Stage 3/4 Endometriosis and Healthy Controls. *Sci Rep*. 2019;9(1):2204. doi:10.1038/s41598-019-39700-6

16. Akiyama K, Nishioka K, Khan KN, Tanaka Y, Mori T, Nakaya T, Kitawaki J. Molecular detection of microbial colonization in cervical mucus of women with and without endometriosis. *Am J Reprod Immunol*. 2019;82(2). doi:10.1111/aji.13147

17. Wei W, Zhang X, Tang H, Zeng L, Wu R. Microbiota composition and distribution along the female reproductive tract of women with endometriosis. *Ann Clin Microbiol Antimicrob*. 2020;19(1):15. doi:10.1186/s12941-020-00356-0

18. Hernandes C, Silveira P, Rodrigues Sereia AF, Christoff AP, Mendes H, Valter de Oliveira LF, Podgaec S. Microbiome Profile of Deep Endometriosis Patients: Comparison of Vaginal Fluid, Endometrium and Lesion. *Diagnostics*. 2020;10(3):163. doi:10.3390/diagnostics10030163

19. Chen S, Gu Z, Zhang W, Jia S, Wu Y, Zheng P, Dai Y, Leng J. Microbiome of the lower genital tract in Chinese women with endometriosis by 16s-rRNA sequencing technique: a pilot study. *Ann Transl Med*. 2020;8(21):1440–1440. doi:10.21037/atm-20-1309

20. Huang L, Liu B, Liu Z, Feng W, Liu M, Wang Y, Peng D, Fu X, Zhu H, Cui Z, et al. Gut Microbiota Exceeds Cervical Microbiota for Early Diagnosis of Endometriosis. *Front Cell Infect Microbiol*. 2021;11:788836. doi:10.3389/fcimb.2021.788836

21. Svensson A, Brunkwall L, Roth B, Orho-Melander M, Ohlsson B. Associations Between Endometriosis and Gut Microbiota. *Reprod Sci*. 2021;28(8):2367–2377. doi:10.1007/s43032-021-00506-5

22. Shan J, Ni Z, Cheng W, Zhou L, Zhai D, Sun S, Yu C. Gut microbiota imbalance and its correlations with hormone and inflammatory factors in patients with stage 3/4 endometriosis. *Arch Gynecol Obstet*. 2021;304(5):1363–1373. doi:10.1007/s00404-021-06057-z

23. Le N, Cregger M, Brown V, Loret de Mola J, Bremer P, Nguyen L, Groesch K, Wilson T, Diaz-Sylvester P, Braundmeier-Fleming A. Association of microbial dynamics with urinary estrogens and estrogen metabolites in patients with endometriosis. *PLoS ONE*. 2021;16(12):e0261362. doi:10.1371/journal.pone.0261362

24. Wessels JM, Domínguez MA, Leyland NA, Agarwal SK, Foster WG. Endometrial microbiota is more diverse in people with endometriosis than symptomatic controls. *Sci Rep*. 2021;11(1):18877. doi:10.1038/s41598-021-98380-3

25. Lee S-R, Lee J-C, Kim S-H, Oh Y-S, Chae H-D, Seo H, Kang C-S, Shin T-S. Altered Composition of Microbiota in Women with Ovarian Endometrioma: Microbiome Analyses of Extracellular Vesicles in the Peritoneal Fluid. *IJMS*. 2021;22(9):4608. doi:10.3390/ijms22094608

26. Yuan W, Wu Y, Chai X, Wu X. The colonized microbiota composition in the peritoneal fluid in women with endometriosis. *Arch Gynecol Obstet*. 2022;305(6):1573–1580. doi:10.1007/s00404-021-06338-7

27. Kunaseth J, Waiyaput W, Chanchaem P, Sawaswong V, Permpech R, Payungporn S, Sophonsritsuk A. Vaginal microbiome of women with adenomyosis: A case-control study. *PLoS ONE*. 2022;17(2):e0263283. doi:10.1371/journal.pone.0263283

28. Lindheim L, Bashir M, Münzker J, Trummer C, Zachhuber V, Leber B, Horvath A, Pieber TR, Gorkiewicz G, Stadlbauer V, Obermayer-Pietsch B. Alterations in Gut Microbiome Composition and Barrier Function Are Associated with Reproductive and Metabolic Defects in Women with Polycystic Ovary Syndrome (PCOS): A Pilot Study. *PLoS ONE*. 2017;12(1):e0168390. doi:10.1371/journal.pone.0168390

29. Liu R, Zhang C, Shi Y, Zhang F, Li L, Wang X, Ling Y, Fu H, Dong W, Shen J, et al. Dysbiosis of Gut Microbiota Associated with Clinical Parameters in Polycystic Ovary Syndrome. *Front Microbiol*. 2017;8. doi:10.3389/fmicb.2017.00324

30. Torres PJ, Siakowska M, Banaszewska B, Pawelczyk L, Duleba AJ, Kelley ST, Thackray VG. Gut Microbial Diversity in Women With Polycystic Ovary Syndrome Correlates With Hyperandrogenism. *The Journal of Clinical Endocrinology & Metabolism*. 2018;103(4):1502–1511. doi:10.1210/jc.2017-02153

31. Qi X, Yun C, Sun L, Xia J, Wu Q, Wang Y, Wang L, Zhang Y, Liang X, Wang L, et al. Gut microbiota–bile acid–interleukin-22 axis orchestrates polycystic ovary syndrome. *Nat Med*. 2019;25(8):1225–1233. doi:10.1038/s41591-019-0509-0

32. Zeng B, Lai Z, Sun L, Zhang Z, Yang J, Li Z, Lin J, Zhang Z. Structural and functional profiles of the gut microbial community in polycystic ovary syndrome with insulin resistance (IR-PCOS): a pilot study. *Research in Microbiology*. 2019;170(1):43–52. doi:10.1016/j.resmic.2018.09.002

33. Hong X, Qin P, Huang K, Ding X, Ma J, Xuan Y, Zhu X, Peng D, Wang B. Association between polycystic ovary syndrome and the vaginal microbiome: A case‐control study. *Clin Endocrinol*. 2020;93(1):52–60. doi:10.1111/cen.14198

34. Zhou L, Ni Z, Yu J, Cheng W, Cai Z, Yu C. Correlation Between Fecal Metabolomics and Gut Microbiota in Obesity and Polycystic Ovary Syndrome. *Front Endocrinol*. 2020;11:628. doi:10.3389/fendo.2020.00628

35. Eyupoglu ND, Ergunay K, Acikgoz A, Akyon Y, Yilmaz E, Yildiz BO. Gut Microbiota and Oral Contraceptive Use in Overweight and Obese Patients with Polycystic Ovary Syndrome. *The Journal of Clinical Endocrinology & Metabolism*. 2020;105(12):e4792–e4800. doi:10.1210/clinem/dgaa600

36. Liang Y, Ming Q, Liang J, Zhang Y, Zhang H, Shen T. Gut microbiota dysbiosis in polycystic ovary syndrome: association with obesity — a preliminary report. *Can J Physiol Pharmacol*. 2020;98(11):803–809. doi:10.1139/cjpp-2019-0413

37. Chu W, Han Q, Xu J, Wang J, Sun Y, Li W, Chen Z-J, Du Y. Metagenomic analysis identified microbiome alterations and pathological association between intestinal microbiota and polycystic ovary syndrome. *Fertility and Sterility*. 2020;113(6):1286-1298.e4. doi:10.1016/j.fertnstert.2020.01.027

38. Zhou L, Ni Z, Cheng W, Yu J, Sun S, Zhai D, Yu C, Cai Z. Characteristic gut microbiota and predicted metabolic functions in women with PCOS. *Endocrine Connections*. 2020;9(1):63–73. doi:10.1530/EC-19-0522

39. Tu Y, Zheng G, Ding G, Wu Y, Xi J, Ge Y, Gu H, Wang Y, Sheng J, Liu X, et al. Comparative Analysis of Lower Genital Tract Microbiome Between PCOS and Healthy Women. *Front Physiol*. 2020;11:1108. doi:10.3389/fphys.2020.01108

40. Lu C, Wang H, Yang J, Zhang X, Chen Y, Feng R, Qian Y. Changes in Vaginal Microbiome Diversity in Women With Polycystic Ovary Syndrome. *Front Cell Infect Microbiol*. 2021;11:755741. doi:10.3389/fcimb.2021.755741

41. Lüll K, Arffman RK, Sola-Leyva A, Molina NM, Aasmets O, Herzig K-H, Plaza-Díaz J, Franks S, Morin-Papunen L, Tapanainen JS, et al. The Gut Microbiome in Polycystic Ovary Syndrome and Its Association with Metabolic Traits. *The Journal of Clinical Endocrinology & Metabolism*. 2021;106(3):858–871. doi:10.1210/clinem/dgaa848

42. Liang Z, Di N, Li L, Yang D. Gut microbiota alterations reveal potential gut–brain axis changes in polycystic ovary syndrome. *J Endocrinol Invest*. 2021;44(8):1727–1737. doi:10.1007/s40618-020-01481-5

43. Mammadova G, Ozkul C, Yilmaz Isikhan S, Acikgoz A, Yildiz BO. Characterization of gut microbiota in polycystic ovary syndrome: Findings from a lean population. *Eur J Clin Invest*. 2021;51(4). doi:10.1111/eci.13417

44. Insenser M, Murri M, del Campo R, Martínez-García MÁ, Fernández-Durán E, Escobar-Morreale HF. Gut Microbiota and the Polycystic Ovary Syndrome: Influence of Sex, Sex Hormones, and Obesity. *The Journal of Clinical Endocrinology & Metabolism*. 2018;103(7):2552–2562. doi:10.1210/jc.2017-02799

45. He F, Li Y. The gut microbial composition in polycystic ovary syndrome with insulin resistance: findings from a normal‐weight population. *J Ovarian Res*. 2021;14(1):50. doi:10.1186/s13048-021-00799-9

46. Dong S, jiao J, Jia S, Li G, Zhang W, Yang K, Wang Z, Liu C, Li D, Wang X. 16S rDNA Full-Length Assembly Sequencing Technology Analysis of Intestinal Microbiome in Polycystic Ovary Syndrome. *Front Cell Infect Microbiol*. 2021;11:634981. doi:10.3389/fcimb.2021.634981

47. Yang Y-L, Zhou W-W, Wu S, Tang W-L, Wang Z-W, Zhou Z-Y, Li Z-W, Huang Q-F, He Y, Zhou H-W. Intestinal Flora is a Key Factor in Insulin Resistance and Contributes to the Development of Polycystic Ovary Syndrome. *Endocrinology*. 2021;162(10):bqab118. doi:10.1210/endocr/bqab118

48. Zhu X, Li Y, Jiang Y, Zhang J, Duan R, Liu L, Liu C, Xu X, Yu L, Wang Q, et al. Prediction of Gut Microbial Community Structure and Function in Polycystic Ovary Syndrome With High Low-Density Lipoprotein Cholesterol. *Front Cell Infect Microbiol*. 2021;11:665406. doi:10.3389/fcimb.2021.665406

49. Chen F, Chen Z, Chen M, Chen G, Huang Q, Yang X, Yin H, Chen L, Zhang W, Lin H, et al. Reduced stress-associated FKBP5 DNA methylation together with gut microbiota dysbiosis is linked with the progression of obese PCOS patients. *npj Biofilms Microbiomes*. 2021;7(1):60. doi:10.1038/s41522-021-00231-6

50. Hassan S, Kaakinen MA, Draisma H, Zudina L, Ganie MA, Rashid A, Balkhiyarova Z, Kiran GS, Vogazianos P, Shammas C, et al. Bifidobacterium Is Enriched in Gut Microbiome of Kashmiri Women with Polycystic Ovary Syndrome. *Genes*. 2022;13(2):379. doi:10.3390/genes13020379

51. Li G, Liu Z, Ren F, Shi H, Zhao Q, Song Y, Fan X, Ma X, Qin G. Alterations of Gut Microbiome and Fecal Fatty Acids in Patients With Polycystic Ovary Syndrome in Central China. *Front Microbiol*. 2022;13:911992. doi:10.3389/fmicb.2022.911992

52. Yang Z, Fu H, Su H, Cai X, Wang Y, Hong Y, Hu J, Xie Z, Wang X. Multi-omics analyses reveal the specific changes in gut metagenome and serum metabolome of patients with polycystic ovary syndrome. *Front Microbiol*. 2022;13:1017147. doi:10.3389/fmicb.2022.1017147

53. Yin G, Chen F, Chen G, Yang X, Huang Q, Chen L, Chen M, Zhang W, Ou M, Cao M, et al. Alterations of bacteriome, mycobiome and metabolome characteristics in PCOS patients with normal/overweight individuals. *J Ovarian Res*. 2022;15(1):117. doi:10.1186/s13048-022-01051-8

54. Oh HY, Kim B-S, Seo S-S, Kong J-S, Lee J-K, Park S-Y, Hong K-M, Kim H-K, Kim MK. The association of uterine cervical microbiota with an increased risk for cervical intraepithelial neoplasia in Korea. *Clinical Microbiology and Infection*. 2015;21(7):674.e1-674.e9. doi:10.1016/j.cmi.2015.02.026

55. Zhang C, Liu Y, Gao W, Pan Y, Gao Y, Shen J, Xiong H. The direct and indirect association of cervical microbiota with the risk of cervical intraepithelial neoplasia. *Cancer Med*. 2018;7(5):2172–2179. doi:10.1002/cam4.1471

56. Klein C, Gonzalez D, Samwel K, Kahesa C, Mwaiselage J, Aluthge N, Fernando S, West JT, Wood C, Angeletti PC. Relationship between the Cervical Microbiome, HIV Status, and Precancerous Lesions. *mBio*. 2019;10(1):e02785-18. doi:10.1128/mBio.02785-18

57. Wu M, Gao J, Wu Y, Li Y, Chen Y, Zhao F, Li C, Ying C. Characterization of vaginal microbiota in Chinese women with cervical squamous intra-epithelial neoplasia. *Int J Gynecol Cancer*. 2020;30(10):1500–1504. doi:10.1136/ijgc-2020-001341

58. Lee YH, Kang G-U, Jeon SY, Tagele SB, Pham HQ, Kim M-S, Ahmad S, Jung D-R, Park Y-J, Han HS, et al. Vaginal Microbiome-Based Bacterial Signatures for Predicting the Severity of Cervical Intraepithelial Neoplasia. *Diagnostics*. 2020;10(12):1013. doi:10.3390/diagnostics10121013

59. Chao X, Wang L, Wang S, Lang J, Tan X, Fan Q, Shi H. Research of the Potential Vaginal Microbiome Biomarkers for High-Grade Squamous Intraepithelial Lesion. *Front Med*. 2021;8:565001. doi:10.3389/fmed.2021.565001

60. Nieves-Ramírez ME, Partida-Rodríguez O, Moran P, Serrano-Vázquez A, Pérez-Juárez H, Pérez-Rodríguez ME, Arrieta MC, Ximénez-García C, Finlay BB. Cervical Squamous Intraepithelial Lesions Are Associated with Differences in the Vaginal Microbiota of Mexican Women. *Microbiol Spectr*. 2021;9(2):e00143-21. doi:10.1128/Spectrum.00143-21

61. Lin S, Zhang B, Lin Y, Lin Y, Zuo X. Dysbiosis of Cervical and Vaginal Microbiota Associated With Cervical Intraepithelial Neoplasia. *Front Cell Infect Microbiol*. 2022;12:767693. doi:10.3389/fcimb.2022.767693

62. Xia Y, Feng Y, Qin T, Zhao X, Lu J, Ma C. Characteristics of Vaginal Microbiome in Reproductive-Age Females with HPV Infection in Xinjiang, China. *Evidence-Based Complementary and Alternative Medicine*. 2022;2022:1–10. doi:10.1155/2022/7332628

63. Zhang Y, Xu X, Yu L, Shi X, Min M, Xiong L, Pan J, Zhang Y, Liu P, Wu G, Gao G. Vaginal Microbiota Changes Caused by HPV Infection in Chinese Women. *Front Cell Infect Microbiol*. 2022;12:814668. doi:10.3389/fcimb.2022.814668

64. Mitra A, MacIntyre DA, Lee YS, Smith A, Marchesi JR, Lehne B, Bhatia R, Lyons D, Paraskevaidis E, Li JV, et al. Cervical intraepithelial neoplasia disease progression is associated with increased vaginal microbiome diversity. *Sci Rep*. 2015;5(1):16865. doi:10.1038/srep16865

65. Audirac-Chalifour A, Torres-Poveda K, Bahena-Román M, Téllez-Sosa J, Martínez-Barnetche J, Cortina-Ceballos B, López-Estrada G, Delgado-Romero K, Burguete-García AI, Cantú D, et al. Cervical Microbiome and Cytokine Profile at Various Stages of Cervical Cancer: A Pilot Study. *PLoS ONE*. 2016;11(4):e0153274. doi:10.1371/journal.pone.0153274

66. Łaniewski P, Barnes D, Goulder A, Cui H, Roe DJ, Chase DM, Herbst-Kralovetz MM. Linking cervicovaginal immune signatures, HPV and microbiota composition in cervical carcinogenesis in non-Hispanic and Hispanic women. *Sci Rep*. 2018;8(1):7593. doi:10.1038/s41598-018-25879-7

67. Wang Z, Wang Q, Zhao J, Gong L, Zhang Y, Wang X, Yuan Z. Altered diversity and composition of the gut microbiome in patients with cervical cancer. *AMB Expr*. 2019;9(1):40. doi:10.1186/s13568-019-0763-z

68. Kwon M, Seo S-S, Kim M, Lee D, Lim M. Compositional and Functional Differences between Microbiota and Cervical Carcinogenesis as Identified by Shotgun Metagenomic Sequencing. *Cancers*. 2019;11(3):309. doi:10.3390/cancers11030309

69. Sims TT, Colbert LE, Zheng J, Delgado Medrano AY, Hoffman KL, Ramondetta L, Jazaeri A, Jhingran A, Schmeler KM, Daniel CR, Klopp A. Gut microbial diversity and genus-level differences identified in cervical cancer patients versus healthy controls. *Gynecologic Oncology*. 2019;155(2):237–244. doi:10.1016/j.ygyno.2019.09.002

70. Tango CN, Seo S-S, Kwon M, Lee D-O, Chang HK, Kim MK. Taxonomic and Functional Differences in Cervical Microbiome Associated with Cervical Cancer Development. *Sci Rep*. 2020;10(1):9720. doi:10.1038/s41598-020-66607-4

71. Chen Y, Qiu X, Wang W, Li D, Wu A, Hong Z, Di W, Qiu L. Human papillomavirus infection and cervical intraepithelial neoplasia progression are associated with increased vaginal microbiome diversity in a Chinese cohort. *BMC Infect Dis*. 2020;20(1):629. doi:10.1186/s12879-020-05324-9

72. So KA, Yang EJ, Kim NR, Hong SR, Lee J-H, Hwang C-S, Shim S-H, Lee SJ, Kim TJ. Changes of vaginal microbiota during cervical carcinogenesis in women with human papillomavirus infection. *PLoS ONE*. 2020;15(9):e0238705. doi:10.1371/journal.pone.0238705

73. Xie Y, Feng Y, Li W, Zhan F, Huang G, Hu H, Xiong Y, Tan B, Chen T. Revealing the Disturbed Vaginal Micobiota Caused by Cervical Cancer Using High-Throughput Sequencing Technology. *Front Cell Infect Microbiol*. 2020;10:538336. doi:10.3389/fcimb.2020.538336

74. Wu S, Ding X, Kong Y, Acharya S, Wu H, Huang C, Liang Y, Nong X, Chen H. The feature of cervical microbiota associated with the progression of cervical cancer among reproductive females. *Gynecologic Oncology*. 2021;163(2):348–357. doi:10.1016/j.ygyno.2021.08.016

75. Zhai Q, Zhang W, Zhang Z, Fu Y, Li Y, Wang X, Li L, Meng Y. Characteristics of the Cervicovaginal Microenvironment in Childbearing-Age Women with Different Degrees of Cervical Lesions and HR-HPV Positivity. *Polish Journal of Microbiology*. 2021;70(4):489–500. doi:10.33073/pjm-2021-046

76. Zhang Z, Li T, Zhang D, Zong X, Bai H, Bi H, Liu Z. Distinction between vaginal and cervical microbiota in high-risk human papilloma virus-infected women in China. *BMC Microbiol*. 2021;21(1):90. doi:10.1186/s12866-021-02152-y

77. Kang G-U, Jung D-R, Lee YH, Jeon SY, Han HS, Chong GO, Shin J-H. Dynamics of Fecal Microbiota with and without Invasive Cervical Cancer and Its Application in Early Diagnosis. *Cancers*. 2020;12(12):3800. doi:10.3390/cancers12123800

78. Mao X, Peng X, Pan Q, Zhao X, Yu Z, Xu D. Uterine Fibroid Patients Reveal Alterations in the Gut Microbiome. *Front Cell Infect Microbiol*. 2022;12:863594. doi:10.3389/fcimb.2022.863594

79. Walther-António MRS, Chen J, Multinu F, Hokenstad A, Distad TJ, Cheek EH, Keeney GL, Creedon DJ, Nelson H, Mariani A, Chia N. Potential contribution of the uterine microbiome in the development of endometrial cancer. *Genome Med*. 2016;8(1):122. doi:10.1186/s13073-016-0368-y

80. Lu W, He F, Lin Z, Liu S, Tang L, Huang Y, Hu Z. Dysbiosis of the endometrial microbiota and its association with inflammatory cytokines in endometrial cancer. *Int J Cancer*. 2021;148(7):1708–1716. doi:10.1002/ijc.33428

81. Gressel GM, Usyk M, Frimer M, Kuo DYS, Burk RD. Characterization of the endometrial, cervicovaginal and anorectal microbiota in post-menopausal women with endometrioid and serous endometrial cancers. *PLoS ONE*. 2021;16(11):e0259188. doi:10.1371/journal.pone.0259188

82. Li C, Gu Y, He Q, Huang J, Song Y, Wan X, Li Y. Integrated Analysis of Microbiome and Transcriptome Data Reveals the Interplay Between Commensal Bacteria and Fibrin Degradation in Endometrial Cancer. *Front Cell Infect Microbiol*. 2021;11:748558. doi:10.3389/fcimb.2021.748558

83. Hawkins GM, Burkett WC, McCoy AN, Nichols HB, Olshan AF, Broaddus R, Merker JD, Weissman B, Brewster WR, Roach J, et al. Differences in the microbial profiles of early stage endometrial cancers between Black and White women. *Gynecologic Oncology*. 2022;165(2):248–256. doi:10.1016/j.ygyno.2022.02.021

84. Nené NR, Reisel D, Leimbach A, Franchi D, Jones A, Evans I, Knapp S, Ryan A, Ghazali S, Timms JF, et al. Association between the cervicovaginal microbiome, BRCA1 mutation status, and risk of ovarian cancer: a case-control study. *The Lancet Oncology*. 2019;20(8):1171–1182. doi:10.1016/S1470-2045(19)30340-7

85. Zhou B, Sun C, Huang J, Xia M, Guo E, Li N, Lu H, Shan W, Wu Y, Li Y, et al. The biodiversity Composition of Microbiome in Ovarian Carcinoma Patients. *Sci Rep*. 2019;9(1):1691. doi:10.1038/s41598-018-38031-2

86. Wang Q, Zhao L, Han L, Fu G, Tuo X, Ma S, Li Q, Wang Y, Liang D, Tang M, et al. The differential distribution of bacteria between cancerous and noncancerous ovarian tissues in situ. *J Ovarian Res*. 2020;13(1):8. doi:10.1186/s13048-019-0603-4

87. Morikawa A, Kawabata A, Shirahige K, Akiyama T, Okamoto A, Sutani T. Altered cervicovaginal microbiota in premenopausal ovarian cancer patients. *Gene*. 2022;811:146083. doi:10.1016/j.gene.2021.146083

Table S2. Methodology of composition analysis and sequencing details

| Study ID | Type of sample | Amplified region | Sequencing platform | Database |
| --- | --- | --- | --- | --- |
| Oakley 2008 | Vaginal swabs |  |  |  |
| Ling 2010 | Vaginal swabs | 454 Pyrosequencing(V3) | 454Roche GS-FLX | RDP |
| Srinivasan 2012 | Vaginal swabs | 454 Pyrosequencing(V3-V4) | 454 FLX pyrosequencing | RDP |
| Shipitsyna 2013 | Vaginal samples | 454 Pyrosequencing(V3-V4) | 454-FLX GS-l00 | RDP |
| Dols 2016 | vaginal swab | 16S rRNA V5-V7 | 454 GS-FLXTitanium | RDP |
| Zozaya 2016 | Vaginal | 16S rRNA V4-V6 | 454 FLX pyrosequencing | RDP |
| Chen 2018 | vaginal swab | 16S rRNA V4 | Illumina MiSeq | Greengenes |
| Ceccarani 2019 | vaginal swab | 16S rRNA V3-V4 | Illumina MiSeq | Greengenes |
| Lynch 2019 | vaginal swab | 16S rRNA V3 | Illumina MiSeq | RDP |
| Tao 2019 | vaginal swab | 16S rRNA V1-V2 | Illumina HiSeq 2500 | SILVA |
| Oerlemans 2020 | vaginal lavage fluid | 16S rRNA V4 | Illumina MiSeq | SILVA |
| Pramanick 2021 | vaginal swab | 16S rRNA V3-V4 | Illumina MiSeq | SILVA |
| Deng 2022 | vaginal swab | 16S rRNA V4 | Illumina Hiseq™ |  |
| Khan 2016 | Endometrial samples,  cystic fluid | Metagenome | Illumina Miseq |  |
| Ata 2019 | stool samples Vaginal samples Cervical samples | 16S rRNA V3-V4 | Illumina MiSeq | RDP |
| Akiyama 2019 | Cervical mucus | 16S rRNA V5-V6 | Illumina MiSeq | Greengenes |
| Wei 2020 | lower third of vagina (CL)  posterior vaginal fornix (CU)  cervical mucus (CV)  endometrium (ET)  peritoneal fluid (PF) | 16S rRNA V4-V5 | Ion torrent PGM |  |
| Hernandes 2020 | 21 vaginal fluid samples,  18eutopic endometrium,  8 endometriotic lesion | 16S rRNA V3-V4 | Illumina MiSeq | NCBI genomic reference sequences  Taxonomy |
| Chen 2020a | 67 cervical canal samples, 65 posterior fornix samples, 2 endometrial samples | 16S rRNA V3-V4 | Illumina HiSeq2500 | Greengenes |
| Huang 2021 | feces,  cervical mucus,  peritoneal fluid | 16S rRNA | Ion Torrent S5 | Greengenes |
| Svensson 2021 | Stool samples | 16S rRNA V1-V2 | Illumina HiSeq | Greengenes |
| Shan 2021 | feces | 16S rRNA V3-V4 | Illumina MiSeq | RDP |
| Le 2021 | Fecal and vaginal swabs | 16S rRNA V4 | Illumina MiSeq | Greengenes |
| Wessels 2021 | endometrial biopsy | 16S rRNA V3 | Illumina MiSeq | NCBI genomic reference sequences |
| Lee 2021 | peritoneal fluid | 16S rRNA V3-V4 | Illumina MiSeq | Greengenes |
| Yuan 2022 | peritoneal fluid | 16S rRNA V4 | Illumina HiSeq2500 | Greengenes |
| Kunaseth 2022 | vaginal swab | 16S rRNA V3-V4 | Illumina MiSeq | SILVA |
| Lindheim 2017 | Stool samples | 16S rRNA V1-V2 | Illumina MiSeq | Greengenes |
| Liu 2017 | feces | 16S rRNA V3-V4 | Illumina MiSeq | RDP |
| Torres 2018 | rectal swab samples | 16S rRNA V4 | Illumina MiSeq | Greengenes |
| Qi 2019 | Fecal samples | Metagenome | Illumina HiSeq2500 | UniProt protein database  human genome reference (hg18) |
| Zeng 2018 | Stool samples | 16S rRNA V3-V4 | Illumina Miseq PE300 | SILVA |
| Hong 2020 | Vaginal swabs | 16S rRNA V3-V4 | Illumina HiSeq2500 | SILVA  NCBI’s 16S rRNA gene database |
| Zhou 2020a | Fecal samples | 16S rRNA | Illumina MiSeq | Greengenes |
| Eyupoglu 2020 | Fecal samples | 16S rRNA V3-V4 | Illumina MiSeq | Greengenes |
| Liang 2020 | Stool samples | 16S rRNA V3-V4 | Illumina MiSeq | Greengenes |
| Chu 2020 | Fecal samples | Shotgun metagenomic | Illumina HiSeq 4000 | KEGG |
| Zhou 2020b | Fecal samples | 16S rRNA V3-V4 | Illumina Miseq PE300 | SILVA |
| Tu 2020 | vaginal and cervical canal samples | 16S rRNA V3-V4 | Illumina MiSeq | KEGG |
| Lu 2021a | Vaginal swabs | 16S rRNA V4 | Illumina NovaSeq 6000 |  |
| Lüll 2021 | Fecal samples | 16S rRNA V3-V4 | Illumina MiSeq | SILVA |
| Liang 2021 | Fecal samples | 16S rRNA V4 | Illumina MiSeq | Greengenes |
| Mammadova 2021 | Stool samples | 16S rRNA V3-V4 | Illumina MiSeq |  |
| Insenser 2018 | Fecal samples | 16S rRNA V4 | Illumina MiSeq | RDP |
| He 2021 | Stool samples | 16S rRNA V3-V4 | Illumina Miseq PE250 | Greengenes |
| Dong 2021 | Fecal samples | 16S rRNA |  | SILVA |
| Yang 2021 | Stool samples | 16S rRNA V4 | Illumina | Greengenes |
| Zhu 2021 | Fecal samples | 16S rRNA V3-V4 | Illumina Miseq PE300 | KEGG |
| Chen 2021 | Fecal samples | 16S rRNA V3-V4 | Illumina MiSeq | mzCloud |
| Hassan 2022 | Fecal | 16S rRNA V3-V4 | Illumina MiSeq | Greengenes |
| Li 2022 | Fecal | 16S rRNA V3-V4 | Illumina MiSeq | SILVA |
| Yang 2022 | Fecal | Shotgun metagenomics | Illumina Hiseq™ 4000 | KEGG |
| Yin 2022 | Fecal | 16S rRNA | Illumina | SILVA |
| Oh 2015 | Cervical swabs | Pyrosequencing V1-V3 | Roche454 GS Junior | EzTaxon-e |
| Zhang 2018 | Cervical biopsy | 16S rRNA V3-V4 | Illumina HiSeq2500 | Greengenes |
| Klein 2019 | cervical mucus | 16S rRNA V4 | Illumina MiSeq | Greengenes |
| Wu 2020 | vaginal swab | 16S rRNA V3-V4 | Illumina MiSeq |  |
| Lee 2020 | vaginal swab | 16S rRNA V3 | Ion PGM | Greengenes |
| Chao 2021 | vaginal swab | 16S rRNA V4 | Illumina HiSeq 2500 | SILVA |
| Nieves-Ramirez 2021 | vaginal swab | 16S rRNA V3 | Illumina Hiseq 2000 | SILVA |
| Lin 2022 | cervical mucus vaginal swab | 16S rRNA V3-V4 | Illumina MiSeq | EzBioCloud |
| Xia 2022 | vaginal swab | 16S rRNA V3-V4 | Illumina NovaSeq 6000 | SILVA |
| Zhang 2022 | vaginal swab | 16S rDNA (metagenome) | Illumina NovaSeq PE250 | SILVA |
| Mitra 2015 | posterior vaginal fornix swab | 16S rRNA V1-V2 | Illumina MiSeq | RDP |
| Audirac-Chalifour 2016 | Cervical swabs | 16S rRNA V3-V4 | Genome Sequencer Titanium Roche-454 | Greengenes |
| Laniewski 2018 | Vaginal swabs | 16S rRNA V4 | Illumina MiSeq | Greengenes |
| Wang 2019 | Fecal samples | 16S rRNA V4 | Illumina HiSeq2500 | Greengenes |
| Kwon 2019 | Cervical swabs | Shotgun metagenomic sequencing | Illumina HiSeq2500 | NCBI genomic reference sequences |
| Sims 2019 | Stool samples | 16S rRNA V4 | Illumina MiSeq | SILVA |
| Tango 2020 | Cervical swabs | 16S rRNA | Roche platform  (Roche454 GS-FLX plus, Branford, CT, USA) | EzBioCloud  KEGG |
| Chen 2020b | lateral and posterior fornix | 16S rRNA V3-V4 | Illumina MiSeq | RDP |
| So 2020 | cervicovaginal swab | 16S rRNA V3-V4 | Illumina MiSeq |  |
| Xie 2020 | vaginal swab | 16S rRNA V4 | Illumina MiSeq | KEGG |
| Wu 2021 | cervical mucus | 16S rRNA V4 | Illumina NovaSeq 6000 | Greengenes |
| Zhai 2021 | cervical mucus | 16S rRNA V3-V4 | Ion S5 | SILVA |
| Zhang 2021 | cervical mucus vaginal swab | 16S rRNA V3-V4 | Illumina MiSeq | RDP KEGG |
| Kang 2020 | fecal swabs | 16S rRNA V4-V5 | Ion PGM | Greengenes |
| Mao 2022 | fresh stool samples | 16S rRNA V1-V9 | dual-channel scanner | human gut bacterial microarrays designed and manufactured by Halgen Ltd |
| Walther-António 2016 | vaginal and cervical (lower genital tract),  endometrial, Fallopian, and ovarian samples | 16S rRNA V3-V5 | Illumina MiSeq |  |
| Lu 2021b | Endometrial tissue | 16S rRNA V3-V4 | Illumina HiSeq2500 | Greengenes |
| Gressel 2021 | Vaginal fornices and Ectocervix swabs Anorectal swabs Endometrial swabs | 16S rRNA V4 | Illumina MiSeq | Greengenes |
| Li 2021 | Endometrial tissue | Pyrosequencing V3-V4 | Illumina MiSeq  MGISeq2000 | KEGG |
| Hawkins 2022 | tumor specimens (tissue samples of the uterus or tumor) | 16S rRNA V1-V3 | Ion Torrent NGS | Greengenes |
| Nené 2019 | cervical smear samples | 16S rRNA V1-V3 | Illumina HiSeq2500 | RDP |
| Zhou 2019 | normal distal fallopian tube tissues ovarian tumor tissues | 16S rRNA V3-V4 | Illumina MiSeq | RDP |
| Wang 2020 | ovarian tissue samples | 16S rRNA V3-V4 | Illumina MiSeq | RDP  KEGG |
| Morikawa 2022 | Cervical smear samples | 16S rRNA V1-V2 | Illumina MiSeq | Greengenes |

Table S3. Quality Assessment of the Included Studies in Meta-analysis Using the Newcastle-Ottawa Scale

| Study ID | Case Definition | Representativeness of the Cases | Selection of Controls | Definition of Controls | Comparability of Cases and Controls | Ascertainment of Exposure | Same Method of Ascertainment for Cases and Controls | Nonresponse Rate | Total |
| --- | --- | --- | --- | --- | --- | --- | --- | --- | --- |
| Oakley 2008 | 1 | 1 |  | 1 | 2 | 1 | 1 |  | 7 |
| Ling 2010 | 1 | 1 | 1 | 1 | 2 | 1 | 1 |  | 8 |
| Srinivasan 2012 | 1 | 1 | 1 | 1 | 2 | 1 | 1 |  | 8 |
| Shipitsyna 2013 | 1 | 1 | 1 | 1 | 2 | 1 | 1 |  | 8 |
| Dols 2016 | 1 | 1 |  | 1 | 2 | 1 | 1 |  | 7 |
| Zozaya 2016 | 1 | 1 |  | 1 | 2 | 1 | 1 |  | 7 |
| Chen 2018 | 1 | 1 | 1 | 1 | 2 | 1 | 1 |  | 8 |
| Ceccarani 2019 | 1 | 1 |  | 1 | 2 | 1 | 1 |  | 7 |
| Lynch 2019 | 1 | 1 | 1 | 1 | 2 | 1 | 1 |  | 8 |
| Tao 2019 | 1 | 1 | 1 | 1 | 2 | 1 | 1 |  | 8 |
| Oerlemans 2020 | 1 | 1 |  | 1 | 1 | 1 | 1 |  | 6 |
| Pramanick 2021 | 1 | 1 |  | 1 | 2 | 1 | 1 |  | 7 |
| Deng 2022 | 1 |  | 1 | 1 | 2 | 1 | 1 |  | 7 |
| Khan 2016 | 1 | 1 |  | 1 | 2 | 1 | 1 |  | 7 |
| Ata 2019 | 1 |  | 1 | 1 | 2 | 1 | 1 |  | 7 |
| Akiyama 2019 | 1 |  |  | 1 | 2 | 1 | 1 |  | 6 |
| Wei 2020 | 1 | 1 |  | 1 | 2 | 1 | 1 |  | 7 |
| Hernandes 2020 | 1 |  |  | 1 | 2 | 1 | 1 |  | 6 |
| Chen 2020a | 1 | 1 |  | 1 | 2 | 1 | 1 |  | 7 |
| Huang 2021 | 1 | 1 |  | 1 | 2 | 1 | 1 |  | 7 |
| Svensson 2021 | 1 |  | 1 |  | 2 | 1 | 1 |  | 6 |
| Shan 2021 | 1 | 1 | 1 | 1 | 2 | 1 | 1 |  | 8 |
| Le 2021 | 1 |  |  | 1 | 2 | 1 | 1 |  | 6 |
| Wessels 2021 | 1 | 1 |  | 1 | 2 | 1 | 1 |  | 7 |
| Lee 2021 | 1 |  |  | 1 | 2 | 1 | 1 |  | 6 |
| Yuan 2022 | 1 | 1 |  | 1 | 2 | 1 | 1 |  | 7 |
| Kunaseth 2022 | 1 | 1 | 1 | 1 | 2 | 1 | 1 |  | 8 |
| Lindheim 2017 | 1 | 1 |  | 1 | 2 | 1 | 1 |  | 7 |
| Liu 2017 | 1 | 1 |  | 1 | 2 | 1 | 1 |  | 7 |
| Torres 2018 | 1 |  | 1 | 1 | 2 | 1 | 1 |  | 7 |
| Qi 2019 | 1 | 1 | 1 | 1 | 2 | 1 | 1 |  | 8 |
| Zeng 2018 | 1 | 1 |  | 1 | 2 | 1 | 1 |  | 7 |
| Hong 2020 | 1 | 1 | 1 | 1 | 2 | 1 | 1 |  | 8 |
| Zhou 2020a | 1 | 1 |  | 1 | 2 | 1 | 1 |  | 7 |
| Eyupoglu 2020 | 1 | 1 |  | 1 | 2 | 1 | 1 |  | 7 |
| Liang 2020 | 1 | 1 |  | 1 | 2 | 1 | 1 |  | 7 |
| Chu 2020 | 1 |  |  | 1 | 2 | 1 | 1 |  | 6 |
| Zhou 2020b | 1 | 1 | 1 | 1 | 2 | 1 | 1 |  | 8 |
| Tu 2020 | 1 | 1 | 1 | 1 | 2 | 1 | 1 |  | 8 |
| Lu 2021a | 1 | 1 | 1 | 1 | 2 | 1 | 1 |  | 8 |
| Lüll 2021 | 1 | 1 | 1 | 1 | 2 | 1 | 1 |  | 8 |
| Liang 2021 | 1 |  | 1 | 1 | 2 | 1 | 1 |  | 7 |
| Mammadova 2021 | 1 |  |  | 1 | 2 | 1 | 1 |  | 6 |
| Insenser 2018 | 1 |  |  | 1 | 2 | 1 | 1 |  | 6 |
| He 2021 | 1 | 1 |  | 1 | 2 | 1 | 1 |  | 7 |
| Dong 2021 | 1 | 1 |  | 1 | 2 | 1 | 1 |  | 7 |
| Yang 2021 | 1 |  | 1 | 1 | 2 | 1 | 1 |  | 7 |
| Zhu 2021 | 1 | 1 | 1 | 1 | 2 | 1 | 1 |  | 8 |
| Chen 2021 | 1 | 1 | 1 | 1 | 2 | 1 | 1 |  | 8 |
| Hassan 2022 | 1 | 1 | 1 | 1 | 2 | 1 | 1 |  | 8 |
| Li 2022 | 1 | 1 | 1 | 1 | 2 | 1 | 1 |  | 8 |
| Yang 2022 | 1 |  |  | 1 | 2 | 1 | 1 |  | 6 |
| Yin 2022 | 1 | 1 | 1 | 1 | 2 | 1 | 1 |  | 8 |
| Oh 2015 | 1 | 1 |  | 1 | 2 | 1 | 1 |  | 7 |
| Zhang 2018 | 1 | 1 |  | 1 | 2 | 1 | 1 |  | 7 |
| Klein 2019 | 1 | 1 | 1 | 1 | 2 | 1 | 1 |  | 8 |
| Wu 2020 | 1 |  |  | 1 | 2 | 1 | 1 |  | 6 |
| Lee 2020 | 1 |  |  | 1 | 2 | 1 | 1 |  | 6 |
| Chao 2021 | 1 | 1 | 1 | 1 | 2 | 1 | 1 |  | 8 |
| Nieves-Ramirez 2021 | 1 | 1 | 1 | 1 | 2 | 1 | 1 |  | 8 |
| Lin 2022 | 1 |  | 1 | 1 | 2 | 1 | 1 |  | 7 |
| Xia 2022 | 1 | 1 | 1 | 1 | 2 | 1 | 1 |  | 8 |
| Zhang 2022 | 1 | 1 | 1 | 1 | 2 | 1 | 1 |  | 8 |
| Mitra 2015 | 1 |  | 1 | 1 | 2 | 1 | 1 |  | 7 |
| Audirac-Chalifour 2016 | 1 |  |  | 1 | 2 | 1 | 1 |  | 6 |
| Laniewski 2018 | 1 | 1 | 1 | 1 | 2 | 1 | 1 |  | 8 |
| Wang 2019 | 1 | 1 |  |  | 2 | 1 | 1 |  | 6 |
| Kwon 2019 | 1 | 1 |  | 1 | 2 | 1 | 1 |  | 7 |
| Sims 2019 | 1 | 1 | 1 | 1 | 2 | 1 | 1 |  | 8 |
| Tango 2020 | 1 | 1 |  | 1 | 2 | 1 | 1 |  | 7 |
| Chen 2020b | 1 | 1 |  | 1 | 2 | 1 | 1 |  | 7 |
| So 2020 | 1 |  | 1 | 1 | 1 | 1 | 1 |  | 6 |
| Xie 2020 | 1 |  | 1 | 1 | 2 | 1 | 1 |  | 7 |
| Wu 2021 | 1 | 1 |  | 1 | 2 | 1 | 1 |  | 7 |
| Zhai 2021 |  | 1 | 1 | 1 | 2 | 1 | 1 |  | 7 |
| Zhang 2021 | 1 |  | 1 | 1 | 2 | 1 | 1 |  | 7 |
| Kang 2020 | 1 | 1 |  | 1 | 2 | 1 | 1 |  | 7 |
| Mao 2022 | 1 | 1 | 1 | 1 | 2 | 1 | 1 |  | 8 |
| Walther-António 2016 | 1 |  |  | 1 | 2 | 1 | 1 |  | 6 |
| Lu 2021b | 1 | 1 |  | 1 | 2 | 1 | 1 |  | 7 |
| Gressel 2021 | 1 | 1 |  | 1 | 2 | 1 | 1 |  | 7 |
| Li 2021 | 1 | 1 |  | 1 | 2 | 1 | 1 |  | 7 |
| Hawkins 2022 |  | 1 |  | 1 | 2 | 1 | 1 |  | 6 |
| Nené 2019 | 1 |  | 1 | 1 | 2 | 1 | 1 |  | 7 |
| Zhou 2019 | 1 |  |  | 1 | 2 | 1 | 1 |  | 6 |
| Wang 2020 | 1 |  |  | 1 | 2 | 1 | 1 |  | 6 |
| Morikawa 2022 | 1 | 1 | 1 | 1 | 2 | 1 | 1 |  | 8 |

Table S4. Subgroup analyses of studies with PCOS patients on different regions and weight (Fecal samples)

| Alpha diversity metrics | Subgroup analyses | Pooled results | | Heterogeneity | |
| --- | --- | --- | --- | --- | --- |
|  |  | SMD (95% CI) | P values | I^2^ | P values |
| Observed species | Obese | -0.45 [-0.79, -0.10] | (P = 0.01) | I² = 0% | (P = 0.46) |
|  | Non-obese | -0.33 [-0.62, -0.04] | (P = 0.03) | I² = 0% | (P = 0.87) |
|  | Studies from China | -0.39 [-0.78, 0.00] | (P = 0.05) | I² = 66% | (P = 0.002) |
|  | Excluding studies from China | -0.44 [-0.73, -0.14] | (P = 0.004) | I² = 4% | (P = 0.35) |
| Chao1 | Obese | -0.38 [-0.72, -0.04] | (P = 0.03) | I² = 13% | (P = 0.33) |
|  | Non-obese | -0.24 [-0.56, 0.08] | (P = 0.15) | I² = 39% | (P = 0.12) |
|  | Studies from China | -0.35 [-0.68, -0.02] | (P = 0.04) | I² = 74% | (P<0.00001) |
|  | Excluding studies from China | 0.05 [-1.10, 1.20] | (P = 0.93) | I² = 60% | (P = 0.2) |
| Shannon index | Obese | -0.31 [-0.58, -0.04] | (P = 0.03) | I² = 0% | (P = 0.62) |
|  | Non-obese | 0.03 [-0.28, 0.35] | (P = 0.83) | I² = 45% | (P = 0.08) |
|  | Studies from China | -0.29 [-0.51, -0.07] | (P = 0.009) | I² = 56% | (P = 0.004) |
|  | Excluding studies from China | -0.00 [-0.32, 0.32] | (P = 0.99) | I² = 54% | (P = 0.05) |

Table S5. Methodology and findings of the included studies assessing beta diversity

| Disease | Sample | Study_ID | Metric | Analysis | Analysis | Finding |
| --- | --- | --- | --- | --- | --- | --- |
| BV | Vaginal | Ling2010 | Unweighted Unifrac |  |  | sig. difference |
|  |  | Zozaya2016 | Unweighted Unifrac |  |  |  |
|  |  | Chen2018 | Unweighted Unifrac |  | ANOSIM | sig. difference (A-_A+) |
|  |  |  | Unweighted Unifrac |  | ANOSIM | no sig. difference (N-_N+) |
|  |  |  | Weighted Unifrac |  | ANOSIM | no sig. difference (A-_A+) |
|  |  |  | Weighted Unifrac |  | ANOSIM | sig. difference (N-_N+) |
|  |  |  | Bray_Curtis |  | ANOSIM | no sig. difference (A-_A+) |
|  |  |  | Bray_Curtis |  | ANOSIM | sig. difference (N-_N+) |
|  |  | Ceccarani2019 | Weighted Unifrac | PCoA | ADONIS | sig. difference |
|  |  |  | Unweighted Unifrac | PCoA | ADONIS | sig. difference |
|  |  | Lynch2019 | Unweighted Unifrac | PCoA | ADONIS | sig. difference |
|  |  |  | Bray_Curtis | PCoA | ADONIS | sig. difference |
|  |  | Tao2019 |  | PCA |  |  |
|  |  | Oerlemans2020 | Bray_Curtis | PCoA |  |  |
|  |  | Pramanick2021 | Weighted Unifrac | PCoA |  | sig. difference |
|  |  | Deng2022 |  | NMDS |  |  |
| EM | Fecal | Ata2019 | Bray_Curtis | PCoA | ADONIS | no sig. difference |
|  |  | Huang2021 | Bray_Curtis | PCoA | ADONIS | sig. difference |
|  |  | Svensson2021 | Bray_Curtis |  | ADONIS | sig. difference |
|  |  | Shan2021 |  | PCoA |  | sig. difference |
|  |  | Le2021 | Weighted Unifrac | PCoA | ADONIS | no sig. difference |
|  |  |  | Unweighted Unifrac | PCoA | ADONIS | no sig. difference |
|  | Vaginal | Ata2019 | Bray_Curtis | PCoA | ADONIS | no sig. difference |
|  |  | Hernandes2020 | Bray_Curtis | PCoA | ADONIS | no sig. difference |
|  |  | Le2021 | Weighted Unifrac | PCoA | ADONIS | no sig. difference |
|  |  |  | Unweighted Unifrac | PCoA | ADONIS | no sig. difference |
|  | Cervical | Ata2019 | Bray_Curtis | PCoA | ADONIS | no sig. difference |
|  |  | Akiyama2019 | Weighted Unifrac | PCA |  | no sig. difference |
|  |  | Huang2021 | Bray_Curtis | PCoA | ADONIS | no sig. difference |
|  | Cervical and Vaginal | Chen2020a | Bray_Curtis | PCoA | ADONIS | no sig. difference |
|  |  |  | Bray_Curtis-Binary | PCoA | ADONIS | no sig. difference |
|  |  |  | Weighted Unifrac | PCoA | ADONIS | no sig. difference |
|  |  |  | Unifrac-Binary | PCoA | ADONIS | no sig. difference |
|  | ET | Hernandes2020 | Bray_Curtis | PCoA | ADONIS | no sig. difference |
|  |  | Wessels2021 | Bray_Curtis | PCoA | ADONIS | no sig. difference |
|  | PF | Huang2021 |  | PCA |  | no sig. difference |
|  |  | Lee2021 | Bray_Curtis | PCoA | ADONIS | sig. difference |
|  |  | Yuan2022 | Unweighted Unifrac |  |  | sig. difference |
| AM | Vaginal | Kunaseth2022 | Bray_Curtis |  |  | no sig. difference |
|  |  |  | Jaccard |  |  | no sig. difference |
| PCOS | Fecal | Lindheim2017 | Weighted Unifrac | PCoA | ADONIS | no sig. difference |
|  |  |  | Unweighted Unifrac | PCoA | ADONIS | sig. difference |
|  |  | Liu2017 | Bray_Curtis |  | ADONIS |  |
|  |  | Torres2018 | Unweighted Unifrac | PCoA | ADONIS | no sig. difference |
|  |  | Qi2019 | Bray_Curtis |  |  | sig. difference |
|  |  | Zeng2018 | Weighted Unifrac | PCoA | ANOSIM | no sig. difference (without IR) |
|  |  |  | Weighted Unifrac | PCoA | ANOSIM | sig. difference (with IR) |
|  |  | Zhou2020a |  | PCoA |  | no sig. difference |
|  |  | Eyupoglu2020 | Weighted Unifrac | PCoA |  | no sig. difference |
|  |  |  | Unweighted Unifrac | PCoA |  | no sig. difference |
|  |  |  | Jaccard | PCoA |  | no sig. difference |
|  |  |  | Bray_Curtis | PCoA |  | no sig. difference |
|  |  | Liang2020 | Unweighted Unifrac | PCoA | ADONIS | sig. difference |
|  |  | Chu2020 |  | PCA |  | no sig. difference |
|  |  |  |  | PCA |  | no sig. difference (PCOS-LB) |
|  |  |  |  | PCA |  | no sig. difference (PCOS-HB) |
|  |  | Zhou2020b |  | PCoA |  | no sig. difference |
|  |  | Lüll2021 | Weighted Unifrac | PCoA | ADONIS | no sig. difference |
|  |  |  | Unweighted Unifrac | PCoA | ADONIS | no sig. difference |
|  |  | Liang2021 | Weighted Unifrac |  |  | no sig. difference |
|  |  | Mammadova2021 | Weighted Unifrac | PCoA | ADONIS | no sig. difference |
|  |  |  | Unweighted Unifrac | PCoA | ADONIS | no sig. difference |
|  |  |  | Bray_Curtis | PCoA | ADONIS | no sig. difference |
|  |  | Insenser2018 | Jaccard |  |  | no sig. difference |
|  |  | He2021 |  | PCoA |  | no sig. difference |
|  |  | Dong2021 |  |  | PLS-DA | no sig. difference |
|  |  | Yang2021 | Unweighted Unifrac | PCoA |  | sig. difference |
|  |  | Zhu2021 | Bray_Curtis | PCoA | ANOSIM | no sig. difference |
|  |  | Chen2021 |  |  | ADONIS | sig. difference |
|  |  |  |  |  | ADONIS | no sig. difference (PCOS-HB) |
|  |  |  |  |  | ADONIS | no sig. difference (PCOS-LB) |
|  |  | Li2022 |  | PCoA |  | no sig. difference |
|  |  |  |  | NMDS |  | no sig. difference |
|  |  | Yang2022 | Bray_Curtis | PCoA |  | sig. difference |
|  |  | Yin2022 |  | PCoA | ADONIS | no sig. difference (Healthy-HB，PCOS-HB，PCOS-LB) |
|  | Vaginal | Hong2020 | Jaccard | PCoA | ADONIS | sig. difference |
|  |  |  | Unweighted Unifrac | PCoA | ADONIS | sig. difference |
|  |  |  | Weighted Unifrac | PCoA | ADONIS | no sig. difference |
|  |  | Tu2020 |  | PCoA |  | no sig. difference |
|  |  | Lu2021a |  | PCA | ADONIS | no sig. difference |
|  |  |  | Bray_Curtis | PCoA | ADONIS | no sig. difference |
|  |  |  | Jaccard | PCoA | ADONIS | sig. difference |
|  | Cervical | Tu2020 |  | PCoA |  | no sig. difference |
| CIN | Vaginal | Lee2020 |  | PCA |  | no sig. difference |
|  |  | Nieves-Ramirez2021 | Bray_Curtis | PCoA | ADONIS | no sig. difference |
|  |  | Xia2022 | Weighted Unifrac | PCoA |  | no sig. difference (HPV+, LSIL) |
|  |  | Zhang2022 | Bray_Curtis | PCoA |  | no sig. difference (HPV+, CIN) |
|  | Cervical | Lin2022 | Bray_Curtis | PCoA | ADONIS | sig. difference |
|  |  | Zhang2018 | Weighted Unifrac | PCoA |  | no sig. difference (CIN2+ VS CIN1-) |
|  | Vaginal and Cervical | Lin2022 | Bray_Curtis | PCoA | ADONIS | no sig. difference |
| CIN-CCA | Vaginal | Łaniewski2018 | Jaccard | PCoA | ADONIS | no sig. difference (ICC, HGD, LGD, HPV+) |
|  |  |  | Bray_Curtis | PCoA | ADONIS | no sig. difference (ICC, HGD, LGD, HPV+) |
|  |  | Chen2020b | Unweighted Unifrac | PCoA | ANOSIM | sig. difference (HPV-, LSIL) |
|  |  |  | Unweighted Unifrac | PCoA | ANOSIM | no sig. difference (HPV-, HSIL) |
|  |  |  | Unweighted Unifrac | PCoA | ANOSIM | sig. difference (HPV-, CCA) |
|  |  | Xie2020 | Weighted Unifrac | PCoA |  | sig. difference (HC, CIN) |
|  |  |  | Weighted Unifrac | PCoA |  | sig. difference (HC, CCA) |
|  | Cervical | Kwon2019 | Bray_Curtis | PCoA |  | no sig. difference (HC, CIN2/3, CCA) |
|  |  |  | Jaccard | PCoA |  | no sig. difference (HC, CIN2/3, CCA) |
|  |  | Tango2020 | Bray_Curtis |  | ADONIS | no sig. difference (HC, CIN2/3-CC) |
|  |  | Wu2021 | Weighted Unifrac | PCoA | ADONIS | no sig. difference (HPV+, LSIL, HSIL and CCA) |
|  |  | Zhai2021 | Weighted Unifrac |  |  | sig. difference (HC, HPV+) |
|  |  |  | Weighted Unifrac |  |  | sig. difference (HC, LSIL) |
|  |  |  | Weighted Unifrac |  |  | sig. difference (HC, HSIL) |
|  |  |  | Weighted Unifrac |  |  | sig. difference (HC, CCA) |
| CCA | Fecal | Wang2019 | Weighted Unifrac | PCoA |  | sig. difference |
|  |  |  | Bray_Curtis | NMDS |  | sig. difference |
|  |  | Sims2019 | Unweighted Unifrac | PCoA |  | no sig. difference |
|  |  | Kang2020 | Bray_Curtis | PCoA | ADONIS | sig. difference |
|  | Cervical | Audirac-Chalifour2016 | Weighted Unifrac | PCoA |  | sig. difference |
|  |  | Zhang2021 | Unweighted Unifrac |  |  |  |
| UF | Fecal | Mao2022 |  | PCoA | ANOSIM | sig. difference |
|  |  |  |  | NMDS | ANOSIM | sig. difference |
| EC | Fecal | Gressel2021 | Weighted Unifrac | PCoA | ADONIS |  |
|  | Vaginal and Cervical | Walther-António2016 | Unweighted Unifrac |  | ADONIS | no sig. difference |
|  |  | Gressel2021 | Weighted Unifrac | PCoA | ADONIS |  |
|  | ET | Walther-António2016 | Unweighted Unifrac |  | ADONIS | no sig. difference |
|  |  |  | Unweighted Unifrac |  | ADONIS | no sig. difference |
|  |  | Lu2021b | Jaccard |  | PERMDISP | sig. difference |
|  |  |  | Bray_Curtis |  | PERMDISP | no sig. difference |
|  |  |  | Bray_Curtis |  | ADONIS | sig. difference |
|  |  |  | Unweighted Unifrac |  | PERMDISP | no sig. difference |
|  |  |  | Unweighted Unifrac |  | ADONIS | sig. difference |
|  |  |  | Weighted Unifrac |  | PERMDISP | sig. difference |
|  |  | Gressel2021 | Weighted Unifrac | PCoA | ADONIS |  |
|  |  | Li2021 | Unweighted Unifrac | NMDS |  | sig. difference |
|  | OT | Walther-António2016 | Unweighted Unifrac |  | ADONIS | sig. difference |
| OC | Cervical | Morikawa2022 | Bray_Curtis | PCoA |  |  |
|  |  |  | Weighted Unifrac | PCoA |  |  |
|  | OT | Zhou2019 | Unweighted Unifrac | PCoA |  | sig. difference |
|  |  | Wang2020 |  | PCoA |  | sig. difference |

PCoA, principal coordinates analysis; PCA, principal component analysis; NMDS, non-metric multidimensional scaling; ADONIS/PERMANOVA, permutational analysis of variance; ANOSIM, analysis of similarities; PLS-DA = principal least squares discriminant analysis;

Figure S1. Funnel plots assessing publication bias in the meta-analyses of alpha diversity

**
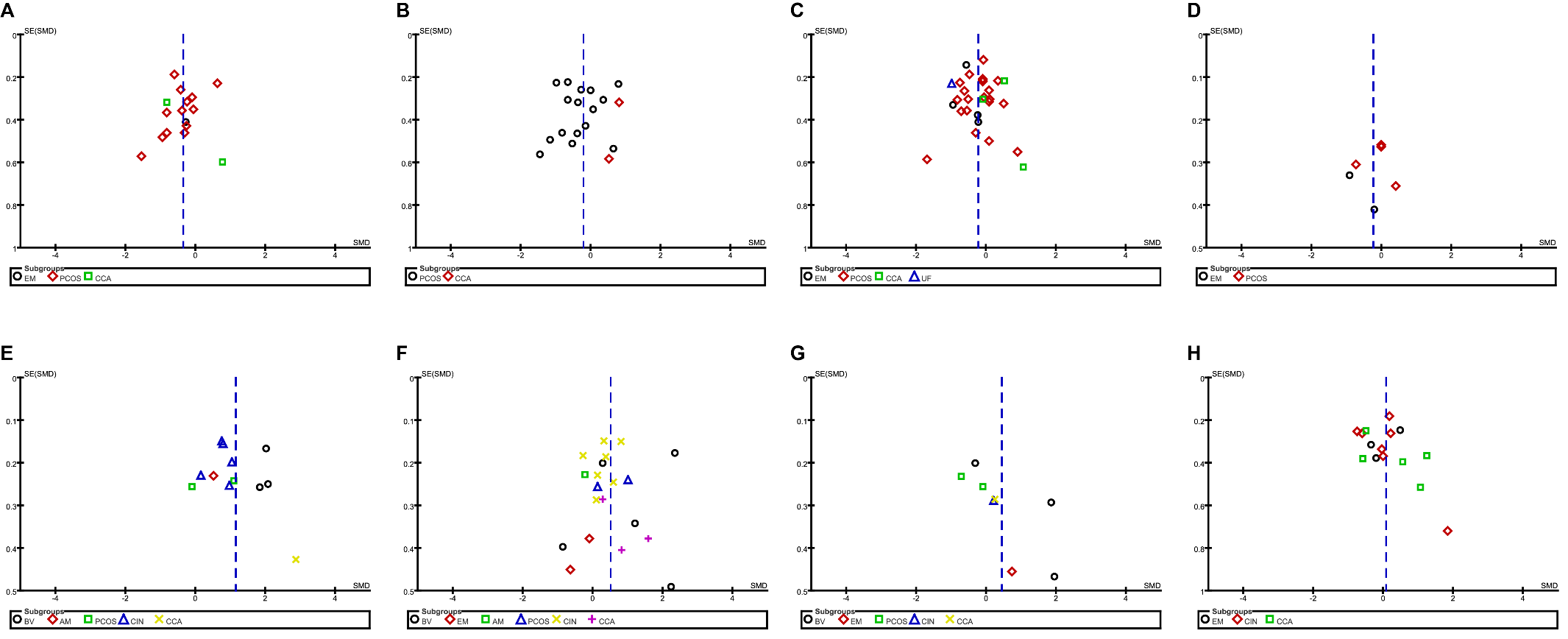
**

**Figure S1. Funnel plots assessing publication bias in the meta-analyses of alpha diversity. (A-D) Observed species, Chao1, Shannon, Simpson index in the gut microbiota; (E-G) Chao1, Shannon, Simpson index in the vaginal microbiota; (H) Shannon index in the cervical microbiota**

Figure S2. Figures for study-level findings of relative abundance of gut and genital microbes


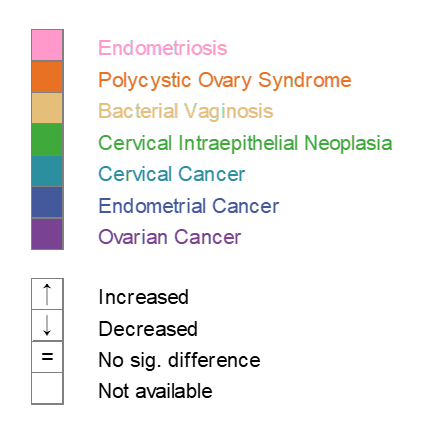


1. **Level: Phylum (in Fecal)**

1. **Level: Family (in Fecal)**

1. **Level: Genus (in Fecal)**

1. **Level: Genus (in Vaginal)**

1. **Level: Genus (in cervix, endometrial tissue, ovarian tissue and peritoneal fluid)**
